# Investigation of the molecular mechanism of *Smilax glabra* Roxb. in treating hypertension based on proteomics and bioinformatics

**DOI:** 10.3389/fphar.2024.1360829

**Published:** 2024-05-09

**Authors:** Xin Yang, Haibing Qian, Changfu Yang, Zhiyuan Zhang

**Affiliations:** Guizhou University of Traditional Chinese Medicine, Guiyang, China

**Keywords:** *Smilax glabra* Roxb., proteomics, high blood pressure, immune cell infiltration, ALDH2

## Abstract

**Background:**

*Smilax glabra* Roxb. (named tufuling in Chinese, SGR) has both medicinal and edible value. SGR has obvious pharmacological activity, especially in anti-inflammation and treating immune system diseases. This study investigated differential protein expression and its relationship with immune infiltration in hypertension treated with SGR using proteomics and bioinformatics.

**Methods:**

N-Nitro L-arginine methyl ester (L-NAME) was used to replicate the hypertension model, with SGR administered by gavage for 4 weeks, and the systolic and diastolic blood pressure in each group of rats was measured using the tail-cuff method every 7 days. Furthermore, enzyme-linked immunosorbent assay (ELISA) was used to determine the serum total cholesterol (TC), triglyceride (TG), low-density lipoprotein cholesterol (LDL-C), and high-density lipoprotein cholesterol (HDL-C) expressions in each group, followed by the detection of protein expression in rat liver samples using the tandem mass tag (TMT) technique. Additionally, hub targets were output using Cytoscape 3.9.1 software, and ALDH2 expression in the liver and serum in each group of rats was detected by ELISA. Moreover, R4.3.0 software was used to evaluate the relationship between acetaldehyde dehydrogenase 2 (ALDH2) and immune cells, and ultra-high performance liquid chromatography–tandem mass spectrometry (UHPLC-MS/MS) was performed to identify the components of SGR. Furthermore, the association between components of SGR and ALDH2 was analyzed with molecular docking and LigPlot1.4.5 software.

**Results:**

Compared with the model group (L-NAME), SGR at high and medium doses reduced systolic and diastolic blood pressure while reducing TC, TG, and LDL-C levels and increasing HDL-C levels in hypertensive rats (*p* < 0.05). Moreover, 92 differentially expressed proteins (DEPs) were identified using TMT. These DEPs participated in peroxisome functioning, fatty acid degradation, and other signaling pathways, with ALDH2 being the core target and correlated with various immune cells. In addition, 18 components were determined in SGR, with 8 compounds binding to ALDH2. Molecular docking was performed to confirm that SGR played a role in hypertension based on the combined action of multiple components.

**Conclusion:**

In conclusion, SGR has an antihypertensive effect on L-NAME-induced hypertension, with ALDH2 as its hub target. SGR may regulate neutrophil, regulatory T cell, and other cells’ infiltration by targeting ALDH2, thereby contributing to the treatment of hypertension.

## Graphical Abstract

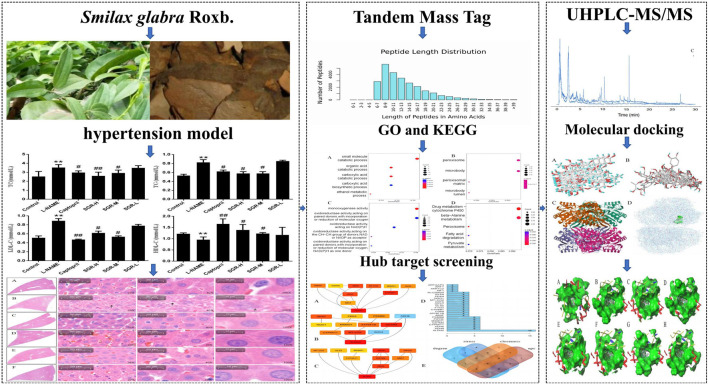

## 1 Introduction

Hypertension is a multi-factorial disease affected by genes, the environment, and lifestyle. Continuous increase in blood pressure can cause vascular endothelial damage, leading to atherosclerosis and cardiovascular diseases (Semenikhina et al., 2024). At present, hypertension is prevalent worldwide; however, its cause is unclear, which is possibly the result of multiple factors combined (Wei et al., 2024). The immune system maintains various physiological activities and is closely related to vascular homeostasis and cardiovascular diseases. The prevention and treatment of hypertension and damage caused by several target organs are recognized globally. Reportedly, the immune system can regulate blood pressure and reduce the damage to target organs. Macrophages, T cells, angiotensin II, and cytokines participate in the occurrence and development of hypertension and its complications through immune mechanisms (Madhur et al., 2021). The activation of the inherent and adaptive immune system causes target-organ damage and dysfunction, and evidently, hypertension is related to abnormal immune activation. As a crucial part of the human system, the immune system interacts and is associated with the circulatory, nervous, and endocrine metabolic systems, which encourages researchers to study traditional Chinese medicine for lowering blood pressure through the immune system.

Traditional Chinese medicine has gradually shown advantages in hypertension treatment. The blood pressure-lowering and target-organ protection mechanisms of traditional Chinese medicine are the centers of research. Studies have found that single-flavored traditional Chinese medicine, traditional Chinese medicine compounds, and traditional Chinese medicine extracts partially affect blood pressure, particularly in preventing damage to several target organs caused by hypertension. Traditional Chinese medicine in hypertension treatment involves multiple targets and pathways to lower blood pressure (Sun et al., 2022). For example, 13 compounds are found in Puerariae Lobatae Radix. (named gegen in Chinese); they regulate multiple targets that can relieve immune inflammatory reactions related to diabetes and hypertension and have significant effects on lowering blood sugar and blood pressure (Wu et al., 2020). For example, Tianma Gouteng decoction and Banxia Baizhu Tianma decoction are traditional Chinese medicine prescriptions for the clinical treatment of hypertension in traditional Chinese medicine (Dong et al., 2020; Jiang et al., 2021). Their main mechanisms of action involve improving endothelial function, inhibiting inflammatory response, reducing oxidative stress, and protecting the heart and blood vessels from lower blood pressure. Astragali Radix (Huangqi), saponin, and polysaccharides mainly provide antioxidant, anti-inflammatory, and anti-apoptosis effects on cardiovascular disease treatment through various pathways (Li et al., 2022). Therefore, we focus on the continuous development of compounds and active ingredients of traditional Chinese medicine with multi-target and -channel prevention and treatment mechanisms of hypertension.

At present, imbalance of the immune regulation function of the body, resulting in a persistent, chronic inflammatory response, is a new focus in the study of hypertension mechanisms. Traditional Chinese medicine can prevent and treat hypertension and target-organ damage through the corresponding immune pathways. *Smilax glabra* Roxb. (SGR) is a traditional Chinese botanical drug, referred to as tufuling in Chinese medicine. Approximately 190 SGR compounds have been isolated and identified, including phenylpropanoids, flavonoids, sterols, organic acids, naphtha, and seven other categories (Shi et al., 2023). SGR has obvious pharmacological activity, especially in anti-inflammation and treating immune system diseases and tumors (Abaidullah et al., 2023; Zhao et al., 2020; Wu et al., 2022). SGR has both medicinal and edible value. In China, a small amount of SGR has been developed as a raw material. Therefore, SGR may help develop functional foods for cardiovascular disease prevention. Therefore, this study aims to investigate the mechanism of SGR in treating hypertension by targeting ALDH2 using proteomics, bioinformatics, and experimental validation strategies.

There are few reports on the use of SGR in hypertension treatment, and the material basis for hypertension treatment is unclear. Therefore, this experiment replicated the L-NAME hypertension model, used proteomics and bioinformatics to find the hub target of hypertension, and analyzed the correlation between the hub target and immune cell infiltration. The chemical composition of SGR was identified by ultra-high performance liquid chromatography–tandem mass spectrometry (UHPLC-MS/MS). The combination of SGR and ALDH2 was analyzed based on molecular docking software (iGEMDOCK and SYBYL 2.1.1). LigPlot1.4.5 software was used to investigate the interaction force between SGR and ALDH2 to clarify the role of hydrogen bonds and hydrophobic forces in docking. The key material basis of SGR for the treatment of hypertension was screened.

## 2 Materials and methods

### 2.1 Experimental instruments

The following instruments were used: a Milli-Q water purifier (Millipore Company, United States); a CT15RE 4°C centrifuge (Hitachi, Japan); a G-560E scroll oscillator (Scientific Industries, United States); the Mettler Toledo ME104E Electronic Balance (China Mettler Toledo Instrument Co., LTD.); a BPH-9162 high-temperature drying oven (Shanghai Heng Scientific Instrument Co., Ltd., China); an ultra-high performance liquid-phase system (Vanquish, Thermo Fisher Scientific); a high-resolution mass spectrometer (Q Exactive Focus, Thermo Fisher Scientific); the 1260 Infinity II high-performance liquid chromatograph (Agilent Corporation, United States); and the EASY-nLC 1000 nA upgraded liquid chromatograph (Thermo Fisher Scientific, United States).

### 2.2 Experimental reagents and plant material

The following materials were used: the TMT10pLex™ isotopic labeling reagent and kit (Thermo Scientific); a protein quantitative assay kit and a gel kit (Kangwei Biotechnology Co., Ltd., China); a 10K ultrafiltration tube (Pall Corporation, United States); formic acid, methanol, and acetonitrile (LC-MS grade, CNW Technologies); L-2-chloro-l-phenylalanine (2-chloro-L-phenylalanine, ≥98%, Shanghai Hengbo Biotechnology Co., Ltd.); and L-nitroarginine (L-NAME, Sigma Corporation, United States). ELISA kits for total cholesterol (TC), triglyceride (TG), low-density lipoprotein cholesterol (LDL-C), and high-density lipoprotein cholesterol (HDL-C) were from Shanghai Enzyme-linked Biotechnology Co., Ltd., China; ELISA kits for ALDH2 was from Shanghai Jiemei Gene Medicine Technology Co., Ltd., China; and BCA protein quantitative assay and SDS-PAGE gel kits were from Beyotime Biotechnology, China.

In April 2022, dried rhizomes of *Smilax glabra* Roxb., a plant belonging to the family Liliaceae, were obtained from Ziyun Miao Buyi Autonomous County, Anshun City, Guizhou Province. The botanical drug was identified by Professor Wei Shenghua of Guizhou University of Traditional Chinese Medicine, and the plant specimens were stored in this university (specimen number: TFL 2022. 04). Captopril (25 mg tablet) was obtained from Guangdong Pi Di Pharmaceutical Co., Ltd., China.

### 2.3 Experimental animals

A total of 46 adult male specific pathogen-free (SPF) Sprague–Dawley (SD) rats with a body mass of (180 ± 20) g were purchased from the Institute of Zoology of Guizhou University of Chinese Medicine and fed in a normal environment. The ambient temperature was (22 ± 4) °C, with 50%–60% humidity, ventilation, 12 h alternating day and night, and free access to food and water. The animal experiment was approved by the Animal Ethics Committee of Guizhou University of Traditional Chinese Medicine (ethical approval number 20220114). The Guide for the Care and Use of Laboratory Animals published by the National Research Council was followed in this work.

### 2.4 *Smilax glabra* Roxb. extraction

SGR was extracted according to a previous report (Shi et al., 2020). In brief, the dried rhizome and root of SGR were pulverized to obtain small pieces (40–60 mm). In a round bottom flask, 300 g of the small pieces was extracted with distilled water (3000 mL) at 80°C for 2 h. After filtering the solution, the resulting residue was decocted for 2 h with 3,000 mL of distilled water. The extraction process was repeated twice, and the extract was collected. After high-speed refrigerated centrifugation at 3,000 rpm for 10 min, the supernatant was collected. The extract was concentrated using a rotary evaporator and freeze-dried. The freeze-dried powder was stored in glass vials at −80°C until required.

### 2.5 Database and software

The database and software required in the experiment are shown in Supplementary Table SA1. The relevant software applications have been authorized.

### 2.6 UHPLC-MS/MS analysis

The extract yield was 129 mg water extract/g dry weight of the rhizome and root of SGR. For UHPLC analysis, a 100-mg aliquot of the sample was precisely transferred to an Eppendorf tube. After the addition of 500 μL extracting solution (methanol: water = 4:1, v/v), the sample was centrifuged at 12,000 rpm at 4°C for 15 min, and 500 μL of the supernatant was passed through a 0.22-μm filter membrane. Chromatographic and mass spectrometry (MS) conditions: ultra-high-performance liquid chromatography (Vanquish, Thermo Fisher Scientific) was performed to analyze specimens using a UPLC BEH C18 chromatography column (100 mm × 2.1 mm, 1.7 μm) based on the mobile phase parameters listed in Supplementary Table SA2 (Zhang et al., 2022). Overall, 5 μL of the sample was injected before adding 0.1% formic acid to mobile phases A and B. For primary and secondary MS data collection, high-resolution MS (Q Exactive Focus, Thermo Fisher Scientific) was performed based on the FullScan ddMS2 function. The detailed parameters were as follows: sheath gas flow rate, 30 Arb; aux gas flow rate, 10 Arb; capillary temperature, 350°C; full MS resolution, 70,000; MS/MS resolution, 17,500; collision energy, 15/30/45 in the NCE mode; and spray voltage, 5.5 kV (positive) or −4.0 kV (negative). Positive and negative ion modes were used to analyze the chemical composition of SGR.

### 2.7 Animal grouping and intervention

The hypertension model included 40 male SD rats intragastrically administered with 40 mg/kg N-nitro L-arginine methyl ester (L-NAME) daily. At 4 weeks after model establishment, a systolic pressure of ≥130 mmHg was induced (Efosa et al., 2023). Thirty rats that were successfully screened for molding were divided into the model (L-NAME), positive-drug captopril (15 mg/kg), SGR high-dose (SGR-H), SGR medium-dose (SGR-M), and SGR low-dose (SGR-L) groups, with six rats in each group. According to the Pharmacological Experimental Methodology and reference, the equivalent dose of daily medication is calculated based on an adult weight of 70 kg, which is approximately 6.3 times that of the weight (Fu et al., 2022; Xia et al., 2010). The groups were administered SGR at doses of 232 mg/kg, 116 mg/kg, and 58 mg/kg for the high-, medium-, and low-dose groups, respectively. The clinical dose equivalent to 58 mg/kg is 450 mg/kg of the raw drug (rhizome and root). Additionally, six normotensive rats were selected as the control group. All rats in the normal and model groups received an equal volume of distilled water. Each group was administered drugs for four consecutive weeks (once daily). A balanced indoor environment was maintained during the feeding process, wherein the animals had free access to food and water. The tail-cuff method was used to determine the systolic and diastolic blood pressure of each group of rats every 7 days. After 4 weeks of intervention and treatment, the rats were anaesthetized, and blood was extracted from the abdominal aorta. Liver and kidney tissue samples of each group of rats were taken and placed in 4% polyformaldehyde; they were then used for paraffin embedding, sectioning, and HE staining. The remaining liver tissue was immediately placed in liquid nitrogen for later use.

### 2.8 Detection of indicators in serum

Abdominal aortic blood was injected into ordinary vacuum blood collection tubes. After 30 min, the sample was freeze-centrifuged at 3,500 rpm for 15 min. Then, the serum was collected. ELISA was used to detect the total cholesterol (TC), triglyceride (TG), low-density lipoprotein cholesterol (LDL-C), and high-density lipoprotein cholesterol (HDL-C), and the enzyme marker was used at a specified wavelength. Absorbance (OD value) was determined, and the sample concentration was calculated. The parameters were measured thrice, and the average value was obtained. The experiment was performed according to the manufacturer’s instructions (Drury et al., 2024).

### 2.9 Tandem mass tag analysis of differential expression proteins

The liver sample was taken out from a −80°C refrigerator and ground to powder. The liver tissues of control, model (L-NAME), and SGR high-dose groups were added to four times the volume of cracking buffer for ultrasonic lysis (8 mol/L urea, 1% protease inhibitor). After centrifugation, the supernatant was transferred to a new centrifuge tube, and the protein was quantified using a BCA protein quantitative kit. A sodium dodecyl sulfate–polyacrylamide gel electrophoresis kit was used to detect protein integrity (Chen et al., 2024). Furthermore, 100 μg of the peptide mixture of each sample was labeled using TMT reagent, according to the manufacturer’s instructions (Thermo Fisher Scientific). The marked mixed peptide segments were graded using the Agilent 1260 Infinity II HPLC system, and each sample was separated using the EASY-nLC system. After the sample was separated via chromatography, a Q Exactive Plus mass spectrometer was used for mass spectrometry analysis. Twenty fragment maps were collected after each full scan. The isolated peptide segment was analyzed online via Proteome Discoverer 2.4.

### 2.10 Gene Ontology and Kyoto Encyclopedia of Genes and Genomes analysis

We performed GO enrichment analysis for functional annotation and KEGG enrichment analysis for signaling pathway annotation of differentially expressed proteins (DEPs). GO and KEGG enrichment analyses were carried out based on the clusterProfiler package in R4.3.0. For enrichment analysis according to the target ID, *p* < 0.05 is selected as the threshold.

### 2.11 Hub target screening

We created a protein–protein interaction (PPI) network using the Search Tool for the retrieval of core targets (STRING, http://string.embl.de/) and visualized using Cytoscape 3.9.1 (https://cytoscape.org/) software. “CytoHubba” (a plug-in) was utilized to identify the hub genes in Cytoscape (Qin et al., 2023).

### 2.12 Correlation analysis between characteristic proteins and immune cells

The hypertension sample was retrieved through the Gene Expression Omnibus (GEO, https://www.ncbi.nlm.nih.gov/geo/) database. GSE24752 and GSE75360 were combined as training datasets, with 14 samples in the normal control group and 13 samples in the hypertension group. The ssGSEA algorithm was used to calculate the immunological abundance of a sample in R software. It used the GSVA R package to calculate the abundance of 28 immune cell species in a single sample. We defined that the cutoff criterion for statistical significance was *p-*value <0.05 (Liu et al., 2024). The horizontal coordinate represents the relevant immune characteristic gene, the vertical coordinate represents the type of immune cell, and the color depth in the module represents the strength of the correlation between the immune characteristic gene and the type of immune cell. Blue represents a negative correlation, while red represents a positive correlation.

### 2.13 Expression of ALDH2 in the serum and liver tissue

ALDH2 expression in serum and liver tissue was determined by the ELISA method (according to the instructions). After collecting blood, the sample was centrifuged at 3,500 r/min for 15 min to quickly separate the serum and red blood cells. Then, 1 g of liver tissue and nine times the amount of saline were fully ground and centrifuged at 3,500 r/min for 15 min. In the envelope of ALDH2 antibodies, samples (serum/liver), standard products, and horseradish peroxidase-labeled detection antibodies were added in turn, and they were warmed and thoroughly washed. Staining was performed with the TMB substrate, which transformed into blue under the catalysis of peroxidase and a final yellow color under the action of acid. The depth of the color was positively correlated with the rat ALDH2 in the sample. The absorbance (OD value) was measured at a wavelength of 450 nm with an enzyme marker to calculate the sample concentration.

### 2.14 Molecular docking and interaction analysis

Through experimental verification and analysis, ALDH2 is the core target, and the molecular docking (iGEMDOCK v2.1 and SYBYL 2.1.1) analysis of SGR components and ALDH2 is completed using the molecular docking module (Yu et al., 2023). The 3D structures of 18 components identified by SGR were downloaded from PUBMED (https://pubchem.ncbi. nlm. nih.gov/). ALDH2 (PDBID: 5L13) 3D crystal structure was obtained from the Research Collaboratory for Structural Bioinformatics (RCSB). ALDH2 generates active pockets based on the original ligand mode, and other parameters use SYBYL default values. The iGEMDOCK v2.1 tool (http://gemdock.life.nctu.edu.tw/dock/igemdock.php) is an open-source molecular docking software package with simple operation, and its results determine the degree of binding with compounds based on the level of energy. Running iGEMDOCK mainly sets the accuracy and speed of docking. In this study, the fast-docking mode was selected, with default parameters of generation 70, number of solution 2, and general evolutionary method 200. The interaction between SGR chemical composition and ALDH2 is based on the software analysis of LigPlot1.4.5 software, and the hydrogen bond and hydrophobic effect formed are calculated (Dubey et al., 2023).

### 2.15 Data analysis

Each group of experimental data is represented by $\bar{x}$ ± s, and the comparison of the two groups of data is tested by the independent sample *t*-test. The comparison between multiple groups of data adopts single-factor variance analysis, with *p* < 0. 05 indicating statistical significance. The statistical chart is drawn by GraphPad Prism software.

## 3 Results

### 3.1 Effects of SGR on blood pressure in hypertensive rats

**FIGURE 1:**
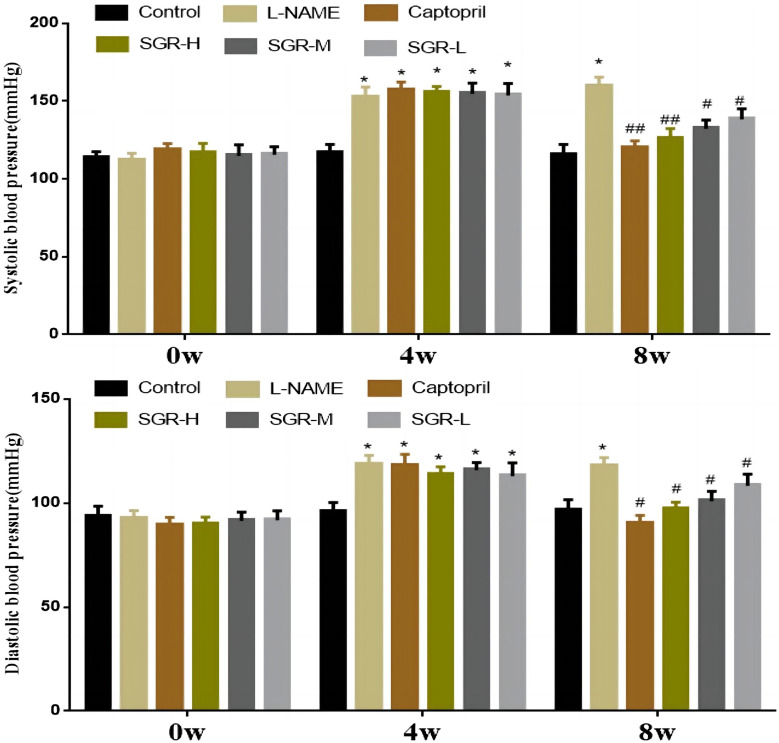
Changes in blood pressure (1 mmHg = 0. 133 kPa). Compared with the normal control group,*p < 0.05 and **p < 0.01; Compared with the model group (L-NAME), ^#^ *p* < 0.05 and ^##^ *p* < 0.01.

In this study, each SD rat was intraperitoneally injected with 40 mg/kg L-NAME for modeling, and 90% of the rats developed hypertension (systolic blood pressure ≥130 mmHg) after 4 w, indicating that the model was reliable. After modeling, systolic and diastolic blood pressure levels were significantly increased in the model group (L-NAME) (*p* < 0.05). SGR-H, SGR-M, SGR-L, and positive (captopril) treatment for 4 weeks could effectively reduce the L-NAME hypertension level (Figure 1). The systolic blood pressure and diastolic blood pressure of rats in SGR-H, SGR-M, SGR-L, and positive (captopril) groups were significantly decreased compared with those in the model group (*p* < 0.05 or *p* < 0.01). The experimental data are shown in Supplementary Table SA3.

### 3.2 Detection of serum lipid indexes in hypertensive rats by *Smilax glabra* Roxb

**FIGURE 2:**
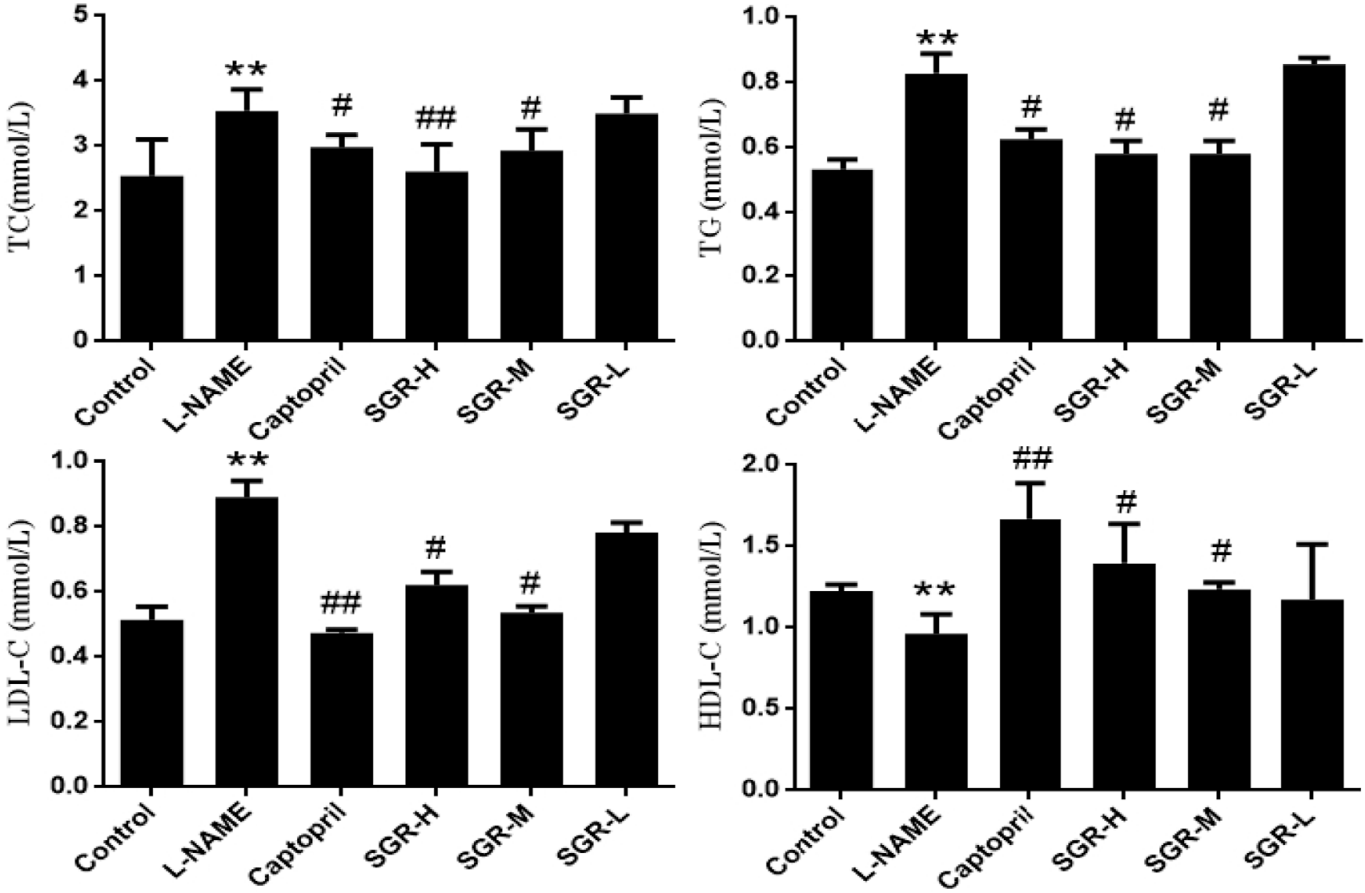
Detection of the serum lipid index. Data are shown as mean ± SD. Compared with the normal control group,*p < 0.05 and **p < 0.05. Compared with the model group (L-NAME), ^#^ *p* < 0.05 and ^##^ *p* < 0.01.

Compared with the normal control group (Figure 2), the levels of TC, TG, and LDL-C were significantly increased in the model group (*p* < 0.05 or *p* < 0.01), while the levels of HDL-C were significantly decreased (*p* < 0.05); compared with the model group (L-NAME), the levels of TC, TG, and LDL-C in captopril, SGR-H, and SGR-M groups were significantly decreased, respectively, and the levels of HDL-C were significantly increased (*p* < 0.05 or *p* < 0.01). The experimental data are shown in Supplementary Table SA4.

### 3.3 Histopathological analysis of the liver and kidney

#### 3.3.1 Histopathological analysis of the liver

**FIGURE 3:**
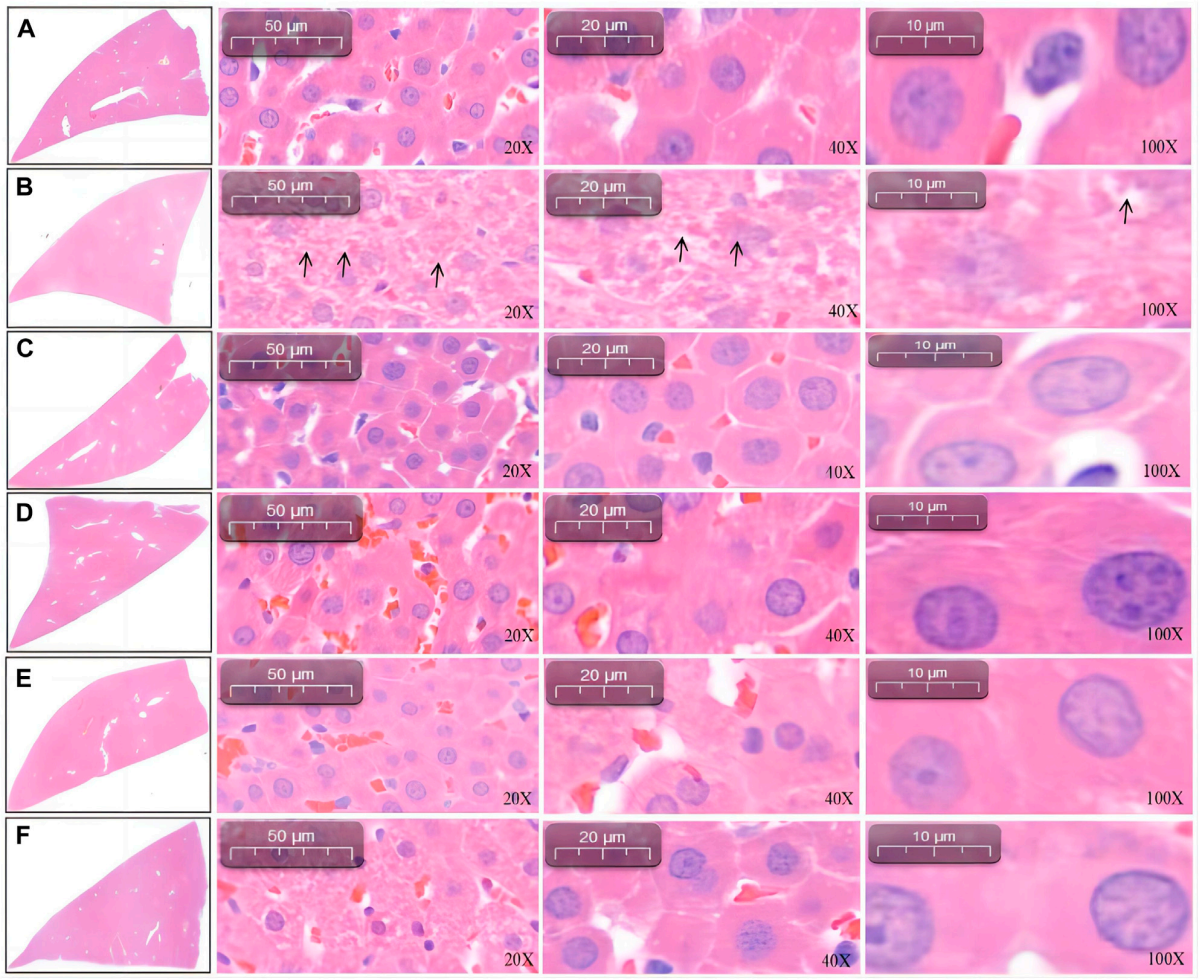
Liver pathology in rats. **(A)** Control. **(B)** Model group (L-NAME). **(C)** Captopril. **(D)** SGR-H. **(E)** SGR-M. **(F)** SGR-L.

Under 20-, 40-, and 100-fold objective lens, liver histopathological staining showed that the liver cells of the blank control group were complete in structure and regular in shape, with normal cell space, most of the nuclei being deeply stained, clear nucleoli, and no degeneration and necrosis (Figure 3A). In contrast, the staining of liver cells in the model group was significantly lighter, and the structure was disorganized, with nuclear pyknosis (Figure 3B). Compared with the model group (L-NAME), the positive-drug (captopril, Figure 3C), SGR-H (Figure 3D), and SGR-M (Figure 3E) groups showed improvement in the abovementioned parameters. However, the SGR-L group (Figure 3F) showed no significant improvement.

#### 3.3.2 Histopathological analysis of the kidney

**FIGURE 4:**
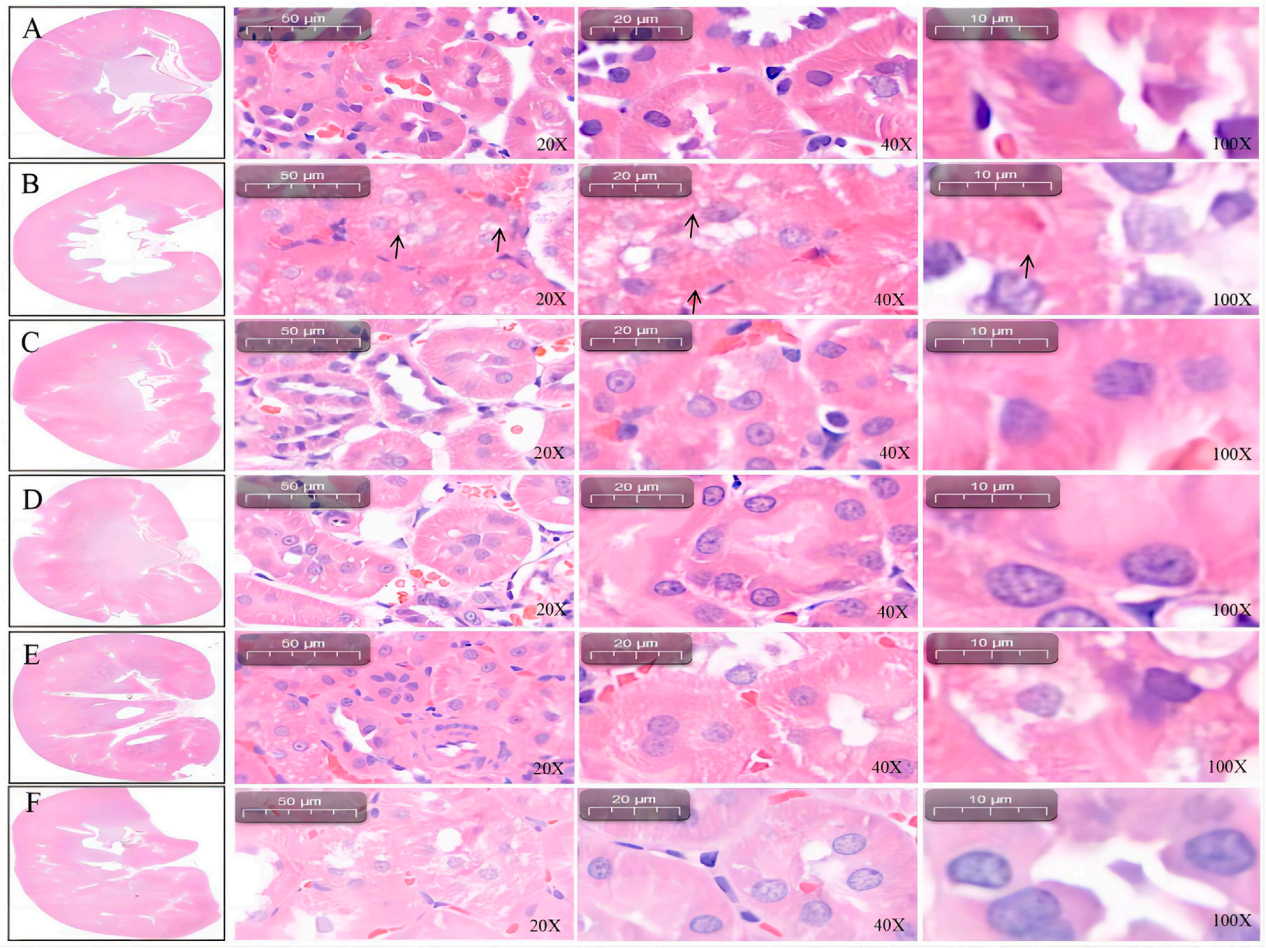
Renal pathology in rats. **(A)** Control. **(B)** Model group (L-NAME). **(C)** Captopril. **(D)** SGR-H. **(E)** SGR-M. **(F)** SGR-L.

Under 20-, 40-, and 100-fold objective lens, renal histopathological staining revealed that the rats in the blank control group had intact glomerular structure and normal balloon proportion. Moreover, the renal tubules were closely packed without inflammatory cell infiltration (Figure 4A). Compared with the blank control group, the model group (L-NAME) had smaller glomeruli, enlarged cystic cavity, disordered renal tubules, and narrow lumen (Figure 4B). Compared with the model group (L-NAME), the positive-drug (captopril, Figure 4C), SGR-H (Figure 4D), and SGR-M (Figure 4E) groups showed improvement in the abovementioned parameters. However, the SGR-L group (Figure 4F) showed no significant improvement.

### 3.4 Mass spectrometric quality control analysis

In mass spectrometry, the fragment ions produced by the peptide segment less than 5 amino acids are small, and sequence identification cannot be effective. If the peptide segment is larger than 20 amino acids, the mass and charge number are high, and it is not suitable for high-energy induced fragmentation. All the peptides identified in this study were in the range of 7–20 amino acids. They are in accordance with the general rules of tryptic hydrolysis and high-energy-induced cleavage fragmentation (Supplementary Figure S1). Supplementary Figure S2 shows the relationship between the molecular weight of protein and coverage identified; the molecular weight of protein is negatively correlated with coverage.

### 3.5 Identification of differentially expressed proteins

**FIGURE 5:**
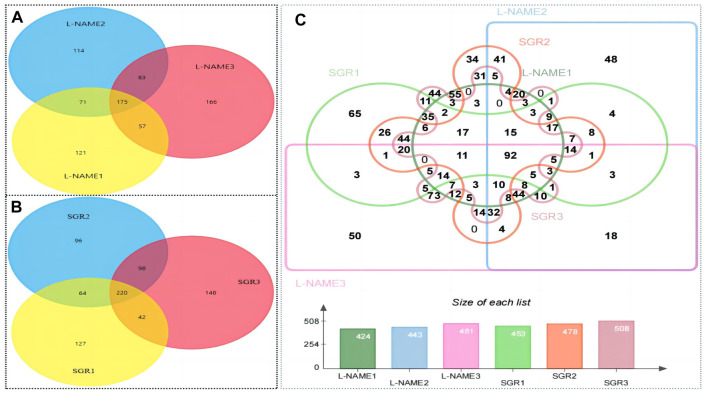
Result of DEP identification. **(A)** Differentially expressed genes in the model group compared with the control group. **(B)** Differentially expressed genes of SGR compared with the model group (L-NAME). **(C)** 92 different proteins in the model group and SGR.

According to the difference in protein abundance levels, the significance of the difference between the experimental group and the control group was assessed by *t*-test. DEPs between different groups were identified according to a fold change >1.2 or <0.85 and a *p* < 0.05. DEPs have two or more specific peptide segments. Compared with the normal control group, 175 DEPs were identified in the model group (Figure 5A). Compared with the model group, 220 DEPs were identified after SGR treatment (Figure 5B). A total of 92 DEPs were identified in the model group and the SGR group (Figure 5C). The 92 DEPs are given in Supplementary Table SA5.

### 3.6 GO and KEGG pathway enrichment analyses of differentially expressed genes

**FIGURE 6:**
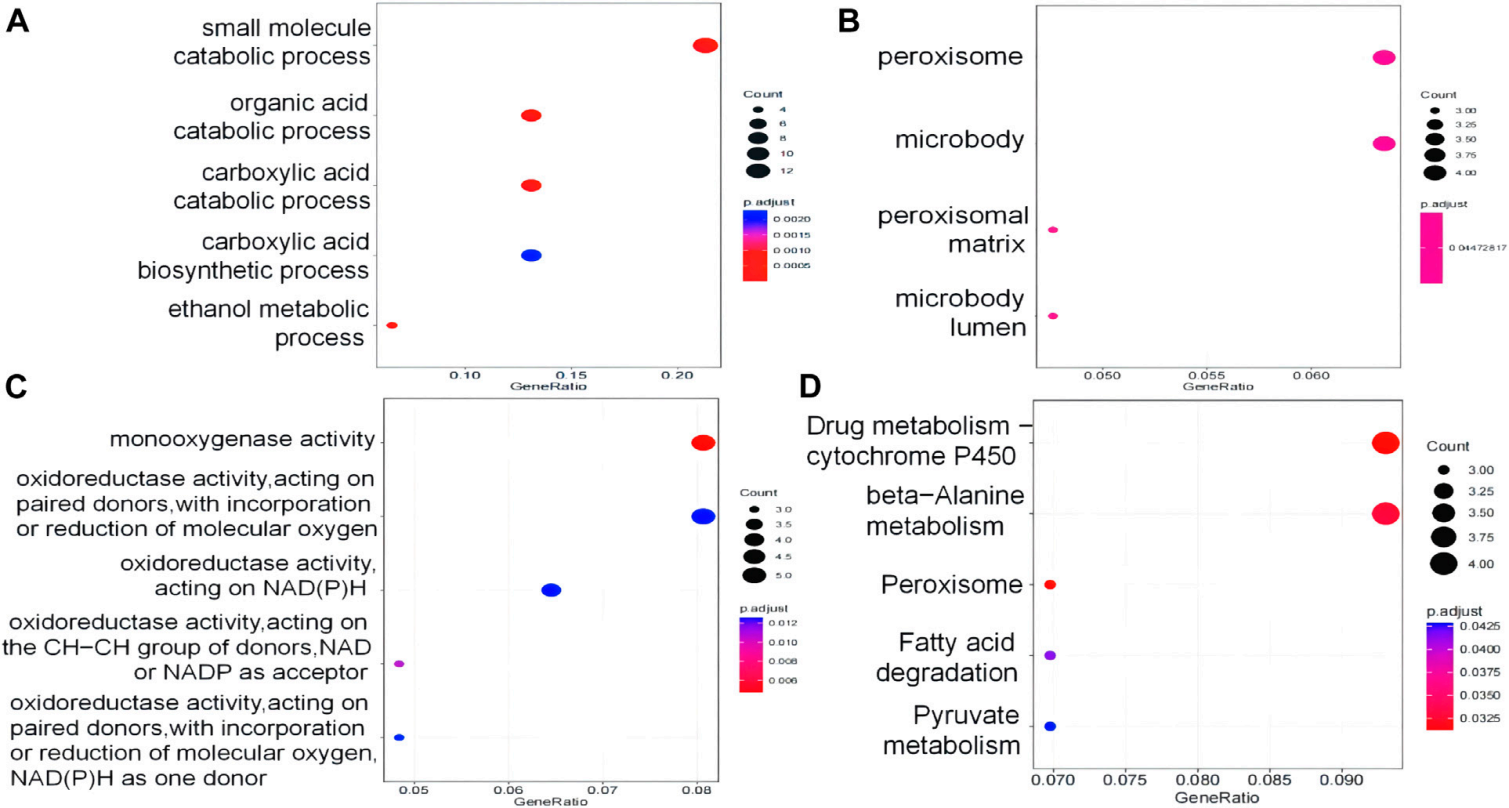
DEPs were analyzed by GO and KEGG. **(A)** Biological process GO terms for DEPs. **(B)** Cellular component GO terms for DEPs. **(C)** Molecular function GO terms for DEPs. **(D)** KEGG pathways for DEPs.

We carried out GO and KEGG enrichment analyses on DEPs. The GO annotations of DEPs consisted of three parts, namely, BP (biological process), MF (molecular function), and CC (cellular component), which were used to analyze the functional enrichment of DEPs. BP terms showed that the DEPs were enriched in “small-molecule catabolic process,” “organic acid catabolic process,” and “carboxylic acid catabolic process” (Figure 6A). MF terms showed that the DEPs were enriched in “monooxygenase activity” (Figure 6B). In terms of CC, the terms “peroxisome,” “microbody,” “membrane microdomain,” and “peroxisomal matrix” were significantly enriched (Figure 6C). KEGG analysis was conducted to determine the relationship between DEPs and signaling pathways. In total, 92 DEPs were mainly involved in “drug metabolism–cytochrome P450,” “peroxisome,” “beta-alanine metabolism,” “fatty acid degradation” and “the pyruvate metabolism signaling pathway” (Figure 6D). GO and KEGG enrichment result analysis is shown in Supplementary Table SA6.

### 3.7 Hub gene identification

**FIGURE 7:**
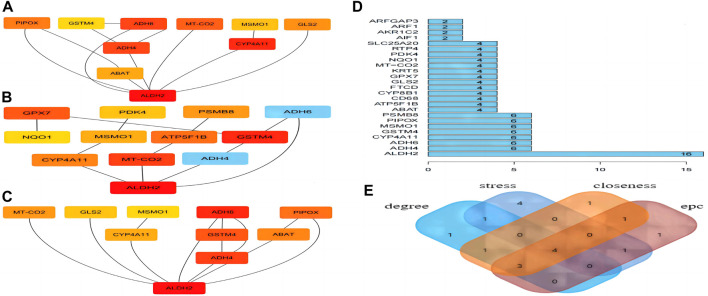
Hub gene analyses. **(A)** EPC. **(B)** Stress. **(C)** Closeness. **(D)** Degree. **(E)** Four hub genes were obtained.

Four algorithms (degree, EPC, stress, and closeness) in “CytoHubba” were used to calculate the weight of each gene in total. Figure 7A shows the hub gene output by EPC. Figure 7B shows the hub gene output by stress. Figure 7C shows the hub gene output by closeness, and Figure 7D shows the hub gene output by degree. Finally, four hub genes (*ALDH2*, *CYP4A11*, *MSMO1*, and *GSTM4*) were obtained (Figure 7E).

### 3.8 Relationship between ALDH2 and immune cell infiltration

**FIGURE 8:**
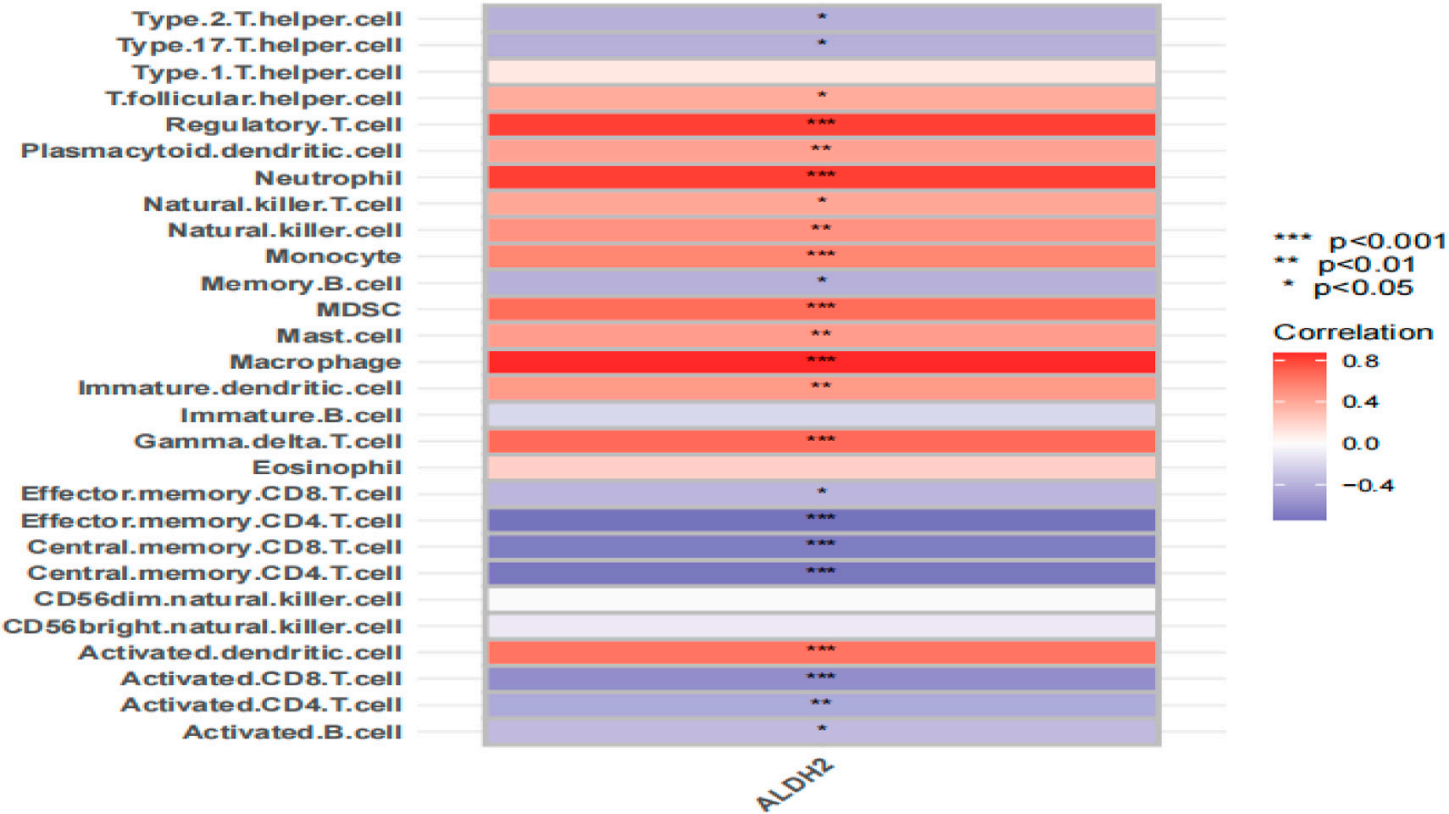
Representative correlation heatmap between the result of the ssGSEA algorithm and ALDH2. Data are shown as mean ± SD. *p < 0.05, **p < 0.01, and ***p < 0.001.

We used the ssGSEA algorithm for analysis. CYP4A11 and GSTM4 had little effect on immune cells (Supplementary Figure S3), and MSMO1 was not found in the GEO dataset. Immune cells had a high correlation with ALDH2. It is found that the *ALDH2* gene is related to various immune cells (Figure 8). ALDH2 is positively correlated with regulatory T cells, neutrophils, macrophages, myeloid-derived suppressor cells, gamma delta T cells, etc. (*p <* 0.001); ALDH2 was negatively correlated with activated CD8 T cells, central memory CD4 T cells, central memory CD8 T cells, etc. (*p* < 0.001). CYP4A11, MSMO1, and GSTM4 do not have a significant relationship with immune cell infiltration and will not be focused on.

### 3.9 ALDH2 expression in serum and liver samples

**FIGURE 9:**
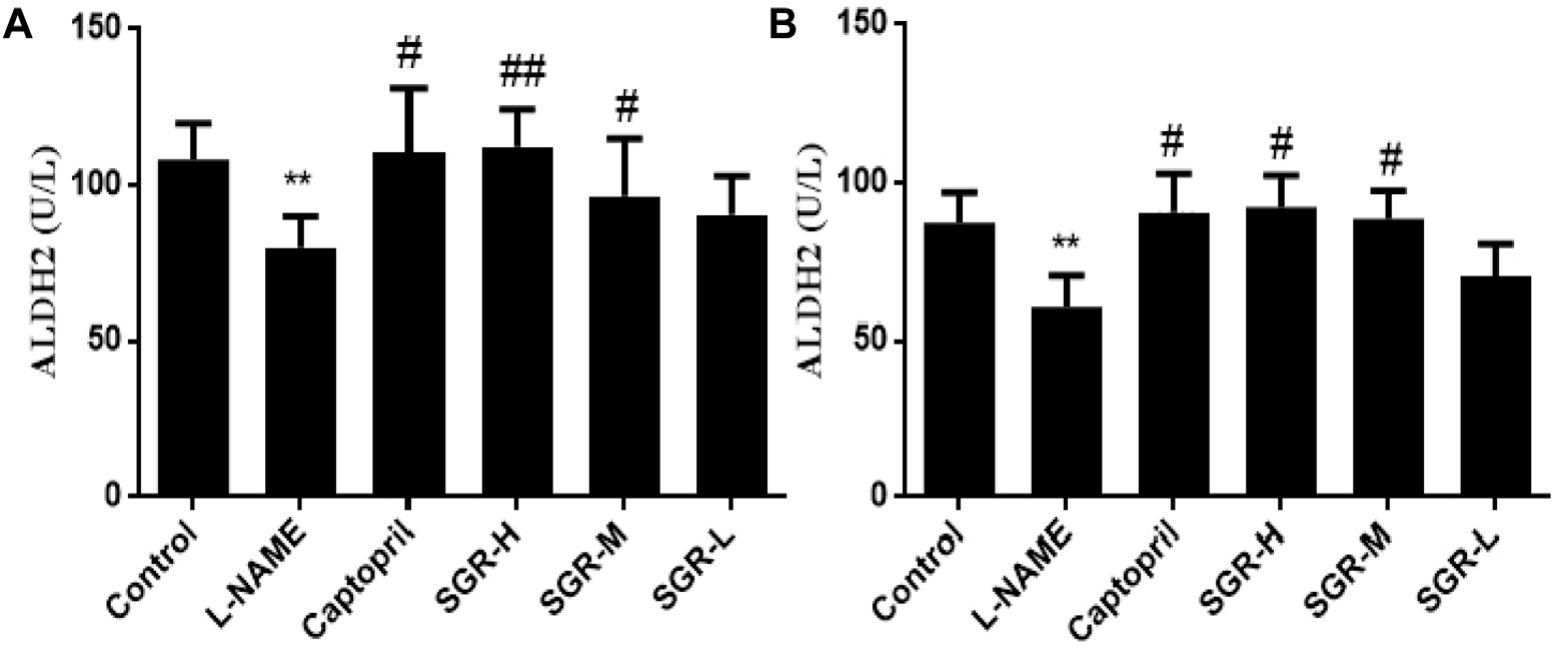
ALDH2 expression in serum and liver samples. **(A)** ALDH2 expression in liver samples. **(B)** ALDH2 expression in serum samples. Compared with the normal control group,*p < 0.05 and **p < 0.05. Compared with the model group (L-NAME), ^#^ *p* < 0.05 and ^##^ *p* < 0.01.

Protein was extracted from liver tissue, and three biological replicates were performed for each group of samples. ALDH2 levels in liver and serum samples were detected by enzyme-linked immunosorbent assay (Figure 9). Compared with the normal control group, the content of ALDH2 in liver and serum samples in the model group (L-NAME) was significantly decreased (*p* < 0.01), which significantly increased after positive-drug, high-dose, and medium-dose SGR intervention administrations (*p* < 0.05 or *p* < 0.01).

### 3.10 Analysis of *Smilax glabra* Roxb. compounds based on UHPLC-MS/MS

**FIGURE 10:**
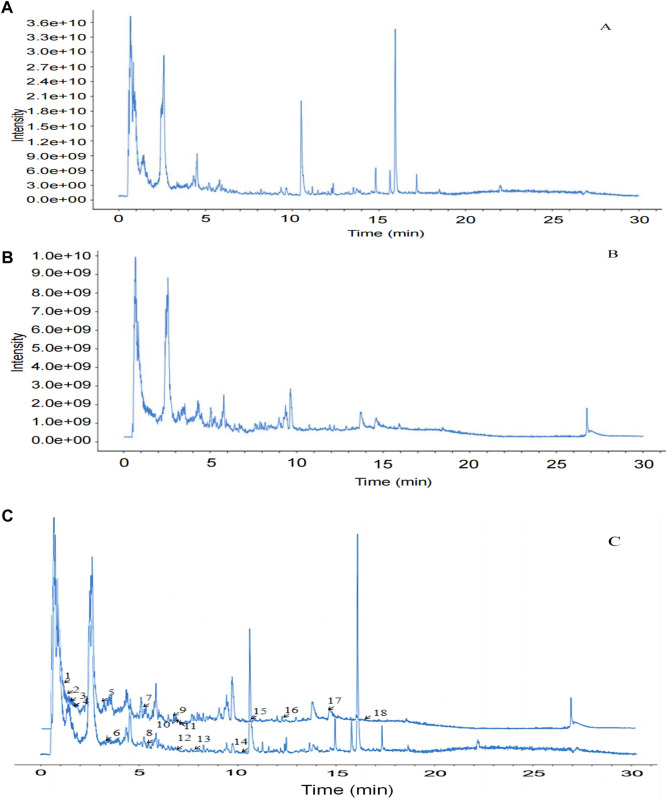
Identification of compounds in water extract solutions of *Smilax glabra* Roxb. by UHPLC-MS/MS. **(A)** Total ion chromatography in positive ion modes for SGR samples as shown. **(B)** Total ion chromatography in negative ion modes for SGR samples as shown. **(C)** Positive and negative ion modes are congruent.

The UHPLC–MS/MS analysis of the water extract was performed to characterize the compounds of SGR. The total ion chromatogram was presented in positive (Figure 10A) and negative (Figure 10B) ion modes. After the preliminary comparative analysis by retention time, MS/MS fragments, and the reported data in references, we found that 18 compounds were found in SGR (Table 1). Figure 10C shows that the positive ion mode and negative ion mode are congruent.

**TABLE 1:** Identification of compounds of *Smilax glabra* Roxb.

NO.	RT	Compound	Formula	MS fragmentation	Class	mzmed
1	0.79	3-Hydroxymethylglutaric acid	C_21_H_24_O_10_	99.045413; 57.034669; 101.024514; 161.044782; 59.013974	Fatty acyls	435.13
2	1.00	Citraconic acid	C_6_H_10_O_5_	85.029118; 71.013723; 129.03832; 57.034664; 101.02462	Fatty acyls	161.05
3	1.06	Maleic acid	C_5_H_6_O_4_	71.013741; 115.004213; 92.683861; 59.014003; 125.633393	Organic acids and derivatives	129.02
4	1.08	2-Isopropylmalic acid	C_4_H_4_O_4_	115.040405; 175.061823; 113.060837; 85.065635; 157.050782	Fatty acyls	115.00
5	3.14	Taxifolin 7-rhamnoside	C_15_H_14_O_6_	449.106458; 259.06008; 269.043351; 125.024193; 287.054231	Alkaloids	289.07
6	3.22	Cianidanol	C_7_H_12_O_5_	139.039037; 123.043436; 147.044267; 165.055433; 291.086353	Phenols	175.06
7	5.04	Quercitrin	C_15_H_14_O_5_	300.024874; 609.140618; 301.032814; 271.022783; 255.02828	Flavonoids	273.08
8	5.57	Isoferulic acid	C_21_H_22_O_11_	177.056017; 163.038708; 149.060337; 145.029117; 117.03355	Phenylpropanoids	449.11
9	6.61	Naringin	C_15_H_10_O_6_	579.169456; 151.003342; 271.06087; 417.156975; 181.050553	Flavonoids	285.04
10	6.65	Hesperetin	C_27_H_32_O_14_	149.023422; 301.035211; 151.002772; 286.046335; 178.997563	Flavonoids	579.17
11	6.78	Naringenin	C_16_H_14_O_6_	271.058824; 151.002846; 119.050539; 107.013599; 93.034225	Flavonoids	301.07
12	6.90	Phloretin	C_10_H_10_O_4_	107.048701; 275.088643; 169.048452; 149.059161; 215.070583	Flavonoids	193.05
13	7.59	Kaempferol	C_21_H_20_O_11_	287.054336; 153.017767; 135.04397; 270.149855; 171.02927	Flavonoids	447.09
14	10.22	Estriol	C_15_H_12_O_5_	289.178707; 271.168678; 159.079888; 119.085963; 107.085309	Steroid	271.06
15	10.45	Neobavaisoflavone	C_18_H_24_O_3_	277.123716; 321.114049; 265.125272; 259.113251; 294.08765	Flavonoids	287.17
16	12.14	Isoxanthohumol	C_20_H_18_O_4_	116.928427; 353.140646; 99.925413; 115.920803; 193.647518	Flavonoids	321.11
17	14.54	Glyceric acid	C_21_H_22_O_5_	105.019216; 106.041133; 72.9929; 75.008343; 59.013576	Carbohydrates and derivatives	353.14
18	16.47	3-Hydroxymethylglutaric acid	C_3_H_6_O_4_	99.045413; 57.034669; 101.024514; 161.044782; 59.013974	Fatty acyls	105.02

### 3.11 Ligand and receptor optimization

**FIGURE 11:**
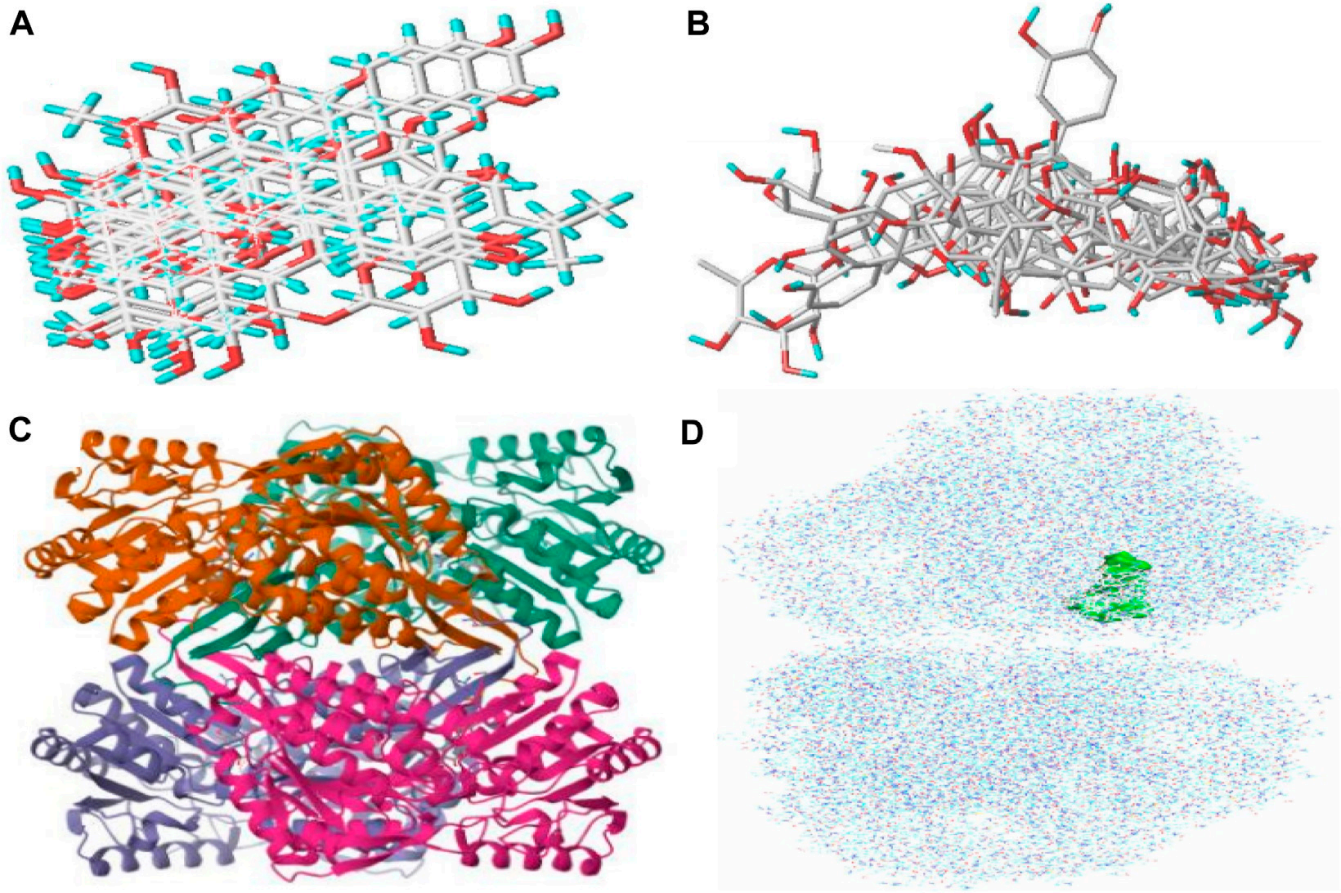
Analysis of the hydrogen bond and hydrophobic action. **(A)** Unoptimized rod model of compounds. **(B)** Optimized rod model of compounds. **(C)** PDB database-downloaded ALDH2 structure. **(D)** Location of the butt pocket.

Ligand optimization is based on SYBYL to optimize SGR active ingredients. Figure 11A is an unoptimized active ingredient stick model (sticks), with a total of 18 ligands. Red represents hydrogen atoms, and blue represents oxygen atoms. The energy minimization calculation based on the Tripos force field of small molecular ligands is carried out, the molecular structure is optimized, and a reasonable conformation is obtained (Figure 11B). Each small molecule is placed in the ligand-binding site of the receptor protein. The ligand configuration and position are optimized so that it has the best binding effect with the receptor, and the best binding conformation is scored. All compounds are sorted according to the scoring, and the 18 ligands are structurally modified to improve the affinity between the ligand and the receptor.

The ALDH2 structure is downloaded from the PDB database (Figure 11C). The resolution is 2.40 Å. Protein chains are displayed in graphical representation, ligands in the form of sticks, and pockets in the form of spheres. The protein is indicated by dark green and brown strips, and the ligand, by sticks. The ligand (A/Q6ZE606) mode is selected to form a pocket by dewatering, adding full hydrogen and electronics through SYBYL. In this molecular docking calculation, it is usually the binding position of small organic molecules. For the X-ray crystal structure of the protein–small-molecule complex, there is a ligand in the pocket, and the green area is the position of the docking pocket, which is the ligand-binding area in the receptor (Figure 11D).

### 3.12 Molecular docking analysis

**FIGURE 12:**
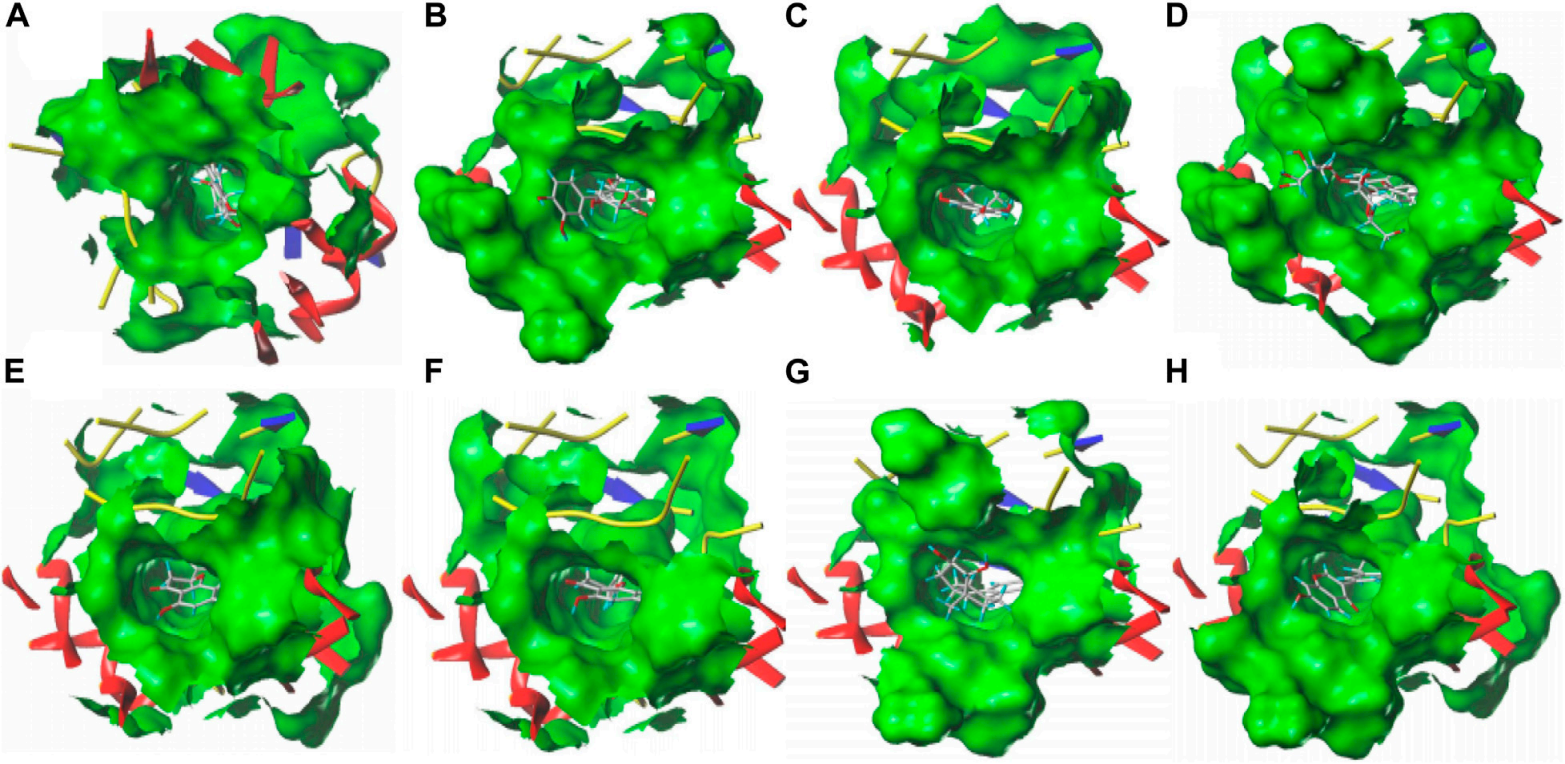
Eight compounds with the ALDH2 docking pocket. **(A)** Cianidanol. **(B)** Taxifolin 7-rhamnoside. **(C)** Phloretin. **(D)** Naringin. **(E)** Hesperetin. **(F)** Naringenin. **(G)** Estriol. **(H)** Neobavaisoflavone.

The combination of SGR and ALDH2 is analyzed through iGEMDOCK and SYBYL 2.1.1 (Table 2). Through SYBYL 2.1.1, it was found that eight compounds have a good combination with ALDH2 (T_Score >5). Through iGEMDOCK, 11 compounds were found to have a good combination with ALDH2 (energy < −100 Kcal/Mol). The two software applications jointly found that eight compounds have a good combination with ALDH2. The binding pockets of the eight compounds are detailed in Figures 12A–H.

**TABLE 2:** Molecular docking analysis.

NO.	Compound	SYBYL (T_Score)	iGEMDOCK(Kcal/mol)
1	3-Hydroxymethylglutaric acid	3.879	−83.3052
2	Citraconic acid	4.445	−71.4994
3	Maleic acid	3.533	−67.1383
4	2-Isopropylmalic acid	4.751	−86.6774
5	Cianidanol	6.515	−119.9860
6	Taxifolin 7-rhamnoside	5.277	−167.2200
7	Isoferulic acid	4.853	−90.3691
8	Phloretin	7.223	−113.8790
9	Quercitrin	−4.642	−132.2950
10	Kaempferol	4.805	−118.4460
11	Naringin	5.502	−161.0290
12	Hesperetin	8.036	−124.2440
13	Naringenin	5.475	−111.0630
14	Estriol	5.031	−117.1780
15	Neobavaisoflavone	6.444	−127.7400
16	Isoxanthohumol	1.806	−127.4020
17	Glyceric acid	3.056	−59.2600
18	3-Hydroxymethylglutaric acid	3.786	−83.6482

### 3.13 Analysis of hydrogen bonds and hydrophobic effects

The two software applications jointly found that eight compounds have a good combination with ALDH2. LigPlot1.4.5 software was used to analyze the interaction force of well-binding protein complexes and make it clear that hydrogen bonds and hydrophobic forces play an important role in docking. The hydrogen bond can display the distance (green line). The eyebrow-like image is hydrophobic. The semicircle is a hydrophobic interaction, with C atoms in black, oxygen atoms in red, and nitrogen atoms in blue. Supplementary Figure S4A shows that cianidanol and ALDH2 combine to form a hydrogen bond (Asp456); Supplementary Figure S4B shows that taxifolin 7-rhamnoside forms one hydrogen bond (Asp457) after combining with ALDH2; Supplementary Figure S4D shows that naringin forms four hydrogen bonds after combining with ALDH2 (Asp457 Cys301 Phe459 Lys127); Supplementary Figure S4E shows that hesperetin and ALDH2 combine to form one hydrogen bond (Asp457); Supplementary Figure S4F shows that naringenin and ALDH2 combine to form one hydrogen bond (Cys302). At the same time, cianidanol, phloretin, hesperetin, naringenin, neobavaisoflavone, taxifolin 7-rhamnoside, naringin, estriol and ALDH2 also have many hydrophobic effects (Table 3).

**TABLE 3:** Main active ingredients that interact with ALDH2.

NO.	Compound	Hydrogen bonding	Hydrophobic action
1	Cianidanol	Asp456	Leu173 Phe296 Phe292 Val120 Phe459 Trp177 Cys303 Cys302 Phe465 Cys301
2	Taxifolin 7-rhamnoside	Asp457	Phe292 Met124 Phe296 Leu173 Phe459 Phe170 Phe465 Trp177 Met174 Cys303 Cys302
3	Phloretin	____	Met124 Phe296 Phe459 Leu173 Phe170 Cys303 Trp177 Cys302 Glu268 Phe465 Met174
4	Naringin	Asp457 Cys301 Phe459 Lys127	Asp147 Gly148 Val120 Phe292 Met124 Phe296 Met174 Trp177 Phe170 Cys303
5	Hesperetin	Asp457	Phe292 Phe296 Val120 Met124 Leu173 Phe459 Cys302 Trp177 Phe465 Glu268
6	Naringenin	Cys302	Phe296 Asp457 Phe459 Cys301 Phe170 Leu173 Trp177 Phe465 Met174 Thr244
7	Estriol	____	Phe170 Phe296 Cys301 Phe459 Asp457 Phe292 Met124 Val120
8	Neobavaisoflavone	____	Met124 Val120 Phe296 Phe459 Asp457 Phe465 Cys301 Phe170 Cys302 Cys303 Asn169

## 4 Discussion

Hypertension refers to a clinical syndrome characterized by an increase in body circulatory arterial blood pressure (systolic/diastolic blood pressure), accompanied by functional or organic damage of the heart, brain, kidney, and other organs. Pathological studies found that the staining of the liver in hypertensive rats was shallow and that the structure was disordered, with nuclear consolidation, obvious edema, and fat degeneration (Ajoolabady et al., 2023). Simultaneously, the glomerulus shrinkage became smaller, the cystic cavity expanded, the renal tubular arrangement was disordered, and the lumen was narrowed (Harrison et al., 2021). Blood pressure in hypertensive rats increased significantly, TC, TG, and LDL-C levels also increased significantly, and HDL-C levels decreased significantly, indicating that the model (L-NAME) was successfully replicated. Long-term elevation in LDL-C and TC levels can harm vascular endothelial cells and their functions, leading to an imbalance in endothelial factors (ET-1/NO), a continuous rise in blood pressure, and an increased risk of hypertension occurrence and progression. Another previous study showed that TG is an important risk factor for hypertension, and controlling TG levels can reduce the incidence of hypertension. This study found that 4 weeks of SGR intervention can effectively reduce systolic and diastolic blood pressure in hypertensive rats, significantly reduce TC, TG, and LDL-C levels, and increase HDL-C levels (Monisa et al., 2023).

This study used proteomics and bioinformatics to screen for core hypertensive targets associated with specific immune cell infiltration. The results indicated that 92 targets of SGR intervention in hypertension were involved in five signaling pathways. Some previous studies report a correlation between fatty acid degradation, beta-alanine metabolism, metabolic signaling pathways, and cardiovascular diseases (Said et al., 2023). Acetate can reduce the risk of cardiovascular diseases by regulating central nervous system function, reducing cholesterol synthesis, and increasing fatty acid oxidation. Acetate can effectively lower blood pressure, improve heart function, and correct lipid metabolism disorders; thus, it influences the prevention and treatment of cardiovascular diseases (Philip et al., 2024). Peroxisome proliferator-activated receptor (PPAR) constitutes a nuclear receptor superfamily. PPAR*α* is mainly related to lipid metabolism. PPAR*γ* has a variety of pathophysiological effects, mainly involving the differentiation of fat cells. The impact of abnormal fatty acid metabolism on hemodynamics has attracted attention, and the findings of several previous studies suggest that hypertension and dyslipidemia have a common genetic and environmental basis (Wang et al., 2023). The findings of this study indicate that the treatment of hypertension with SGR may be related to pathways such as beta-alanine metabolism, fatty acid metabolism, and pyruvate metabolism.

Analysis of protein interaction revealed that acetaldehyde dehydrogenase 2 (ALDH2) is the core target. ALDH2 is a mitochondrial-specific enzyme and one of the most important protective factors in the body. It was widely distributed in tissues of the liver, heart, and brain. Its functions are to prevent lipid peroxidation of membranes by acetaldehyde and inhibit cell apoptosis. ALDH2 can exert antioxidant effects by metabolizing 4-HNE, thereby inhibiting the occurrence and development of hypertension (Zhang et al., 2023a). Previous studies report that ALDH2 deficiency increases oxidative stress in the body and is a susceptibility factor for hypertension (Tanaka et al., 2023). Some previous studies have also found that tALDH2 plays an important role in cardiovascular and nervous systems, tumors, and other diseases and is an important target for preventing and intervening in cardiovascular diseases (Zeng et al., 2023). Immune cell infiltration indicates that the ALDH2 gene is correlated with various immune cells. Mutant ALDH2 enhances the formation of foam cells by affecting the autophagy ability and lipid metabolism of macrophages (Chang et al., 2022). The myeloid-derived suppressor cell (MDSC), regulatory T cells, neutrophils, and other cells play important roles in cardiovascular diseases. Regulatory T cells, also known as suppressive T cells, are a subset of T cells with regulatory functions, including immunosuppressive functions, maintaining self-tolerance and avoiding immune response damage (Alexander and Harrison, 2024). They play an important regulatory role in various immune diseases. MDSC is an anti-inflammatory immune cell characterized by CD11b and Gr-1 expression. It inhibits T-cell activity through hydrogen peroxide, thereby reducing the number and pro-inflammatory phenotype of inflammatory cells in the spleen and kidneys, further regulating the immune system and hypertension (Zhang et al., 2023b). However, there are relatively few reports on the correlation between ALDH2 and these immune cells. This study found that SGR affects the expression of ALDH2 and, through screening, showed that the expression of ALDH2 is correlated with immune cells. However, whether SGR affects the expression of ALDH2 and further affects the expression of immune cells requires further investigation.

SGR has both medicinal and edible value. Eighteen components of SGR were detected by UHPLC-MS/MS, and eight compounds had good binding with ALDH2 through UHPLC-MS/MS and SYBYL 2.1.1. Traditional application of SGR can treat various diseases (such as inflammation, brucellosis, syphilis, and acute and chronic nephritis) and can also serve as an immune modulator and liver protector (Wang et al., 2022; Zhao et al., 2021). Further molecular docking indicated that six compounds (cianidanol, phloretin, hesperetin, naringenin, naringin, and taxifolin 7-rhamnoside) may serve as potential intervention therapy drugs for hypertension protective factors. As the two compounds (neobavaisoflavone and esterol) have not been thoroughly studied to date, they are worthy of further in-depth research. Experiments with animals indicate that cianidanol can lower blood pressure, protect endothelial cells, reduce oxidative stress response, alleviate inflammation, and improve blood lipid distribution (Patanè et al., 2023); phloretin has anti-inflammatory, antioxidant, and blood pressure-lowering effects (Nakhate et al., 2022); hesperetin is a dihydroflavonoid compound with various biological activities, such as antioxidant, anticancer, and lipid-lowering effects (Mu et al., 2022); naringenin has several pharmacological properties. Naringenin can improve obesity, diabetes, and hypertension (Liu et al., 2022); neobavaisoflavone has anti-inflammatory, anticancer, and antioxidant effects (Li et al., 2023; Yuan et al., 2022). However, the impact of neobavaisoflavone on blood pressure has not been explored, necessitating further research. Naringin is a flavanone glycoside with pharmacological effects, including antioxidant, lipid-lowering, anticancer, and inhibition of cytochrome P450 enzymes (Peng et al., 2024; Effat et al., 2024). Previous studies have reported that a long-term increase in the intake of flavonoids can effectively reduce the incidence rate of cardiovascular diseases in the population. Screening of active ingredients indicated that flavonoids from SGR may be the key substance basis for lowering blood pressure. Therefore, this study further developed active ingredients for the treatment of hypertension from SGR, providing targeted inhibitors for the treatment of hypertension.

## 5 Conclusion

In summary, this study found that the levels of TG, TC, and LDL-C in the serum of hypertensive rats were significantly higher than those of the normal control group, whereas the levels of HDL-C were significantly lower than those of the normal control group. L-NAME-induced hypertensive rats exhibited dyslipidemia. Proteomic analysis indicated that SGR alleviates hypertension through multiple pathways and targets, which may be related to pathways such as fatty acid metabolism and pyruvate metabolism. SGR regulates the expression of 92 targets, with ALDH2 being the most core target. Immune cell infiltration analysis showed that the expression of ALDH2 is correlated with various immune cells. Whether SGR can further affect the expression of related immune cells by affecting the expression of ALDH2 is still unclear. Molecular docking analysis indicated that eight compounds had good binding with ALDH2, forming multiple hydrogen bonds. Six compounds play a role in hypertension, whereas two compounds have not been reported to be relevant and require further investigation. Therefore, the focus of this study was on developing active ingredients for the treatment of hypertension from SGR and promoting the clinical application of SGR in the prevention and treatment of hypertension. There were also some shortcomings in this study. *In vivo* experimental verification and analysis of the eight selected compounds have not yet been conducted. The mechanism of action of the eight compounds on hypertension requires further investigation.

## Data Availability

The mass spectrometry proteomics raw datasets generated for this study are publicly available. This data can be found at ProteomeXchange Consortium via the iProX repository: https://www.iprox.cn/page/PSV023.html;?url=1714224166420eTjh (Password:a9ZW).
