# Supplementary material for: Investigation of the molecular mechanism of Smilax glabra Roxb. in treating hypertension based on proteomics and bioinformatics
Source: Front Pharmacol. 2024 May 9;15:1360829. doi: 10.3389/fphar.2024.1360829 (PMC11112092; doi:10.3389/fphar.2024.1360829)
Supplement: Supplementary file 1 [file DataSheet1.doc]

Supplementary Material

# Supplementary Figures and Tables

## Supplementary Figures


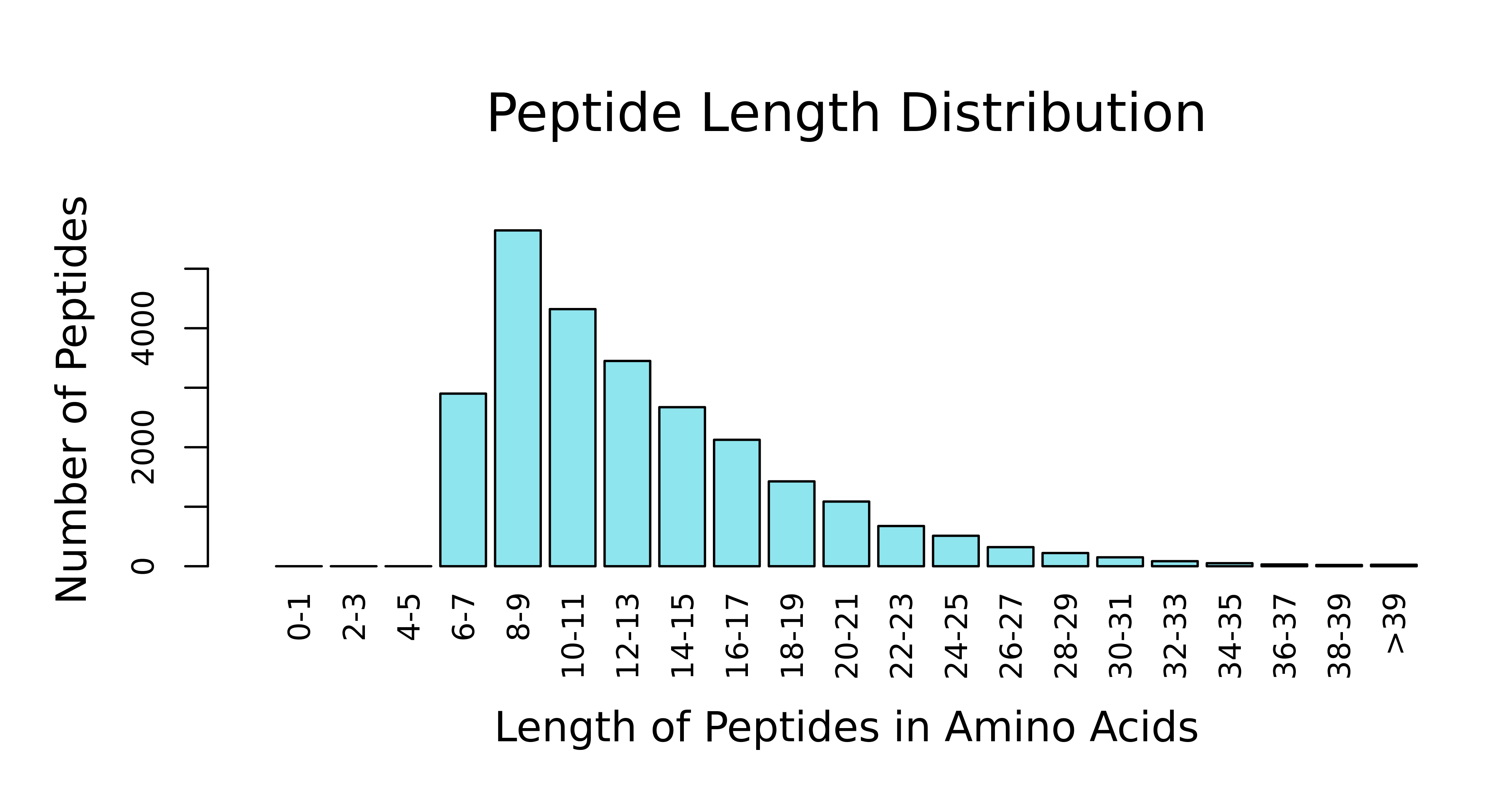


Figure S1.The length distribution of all identified peptides
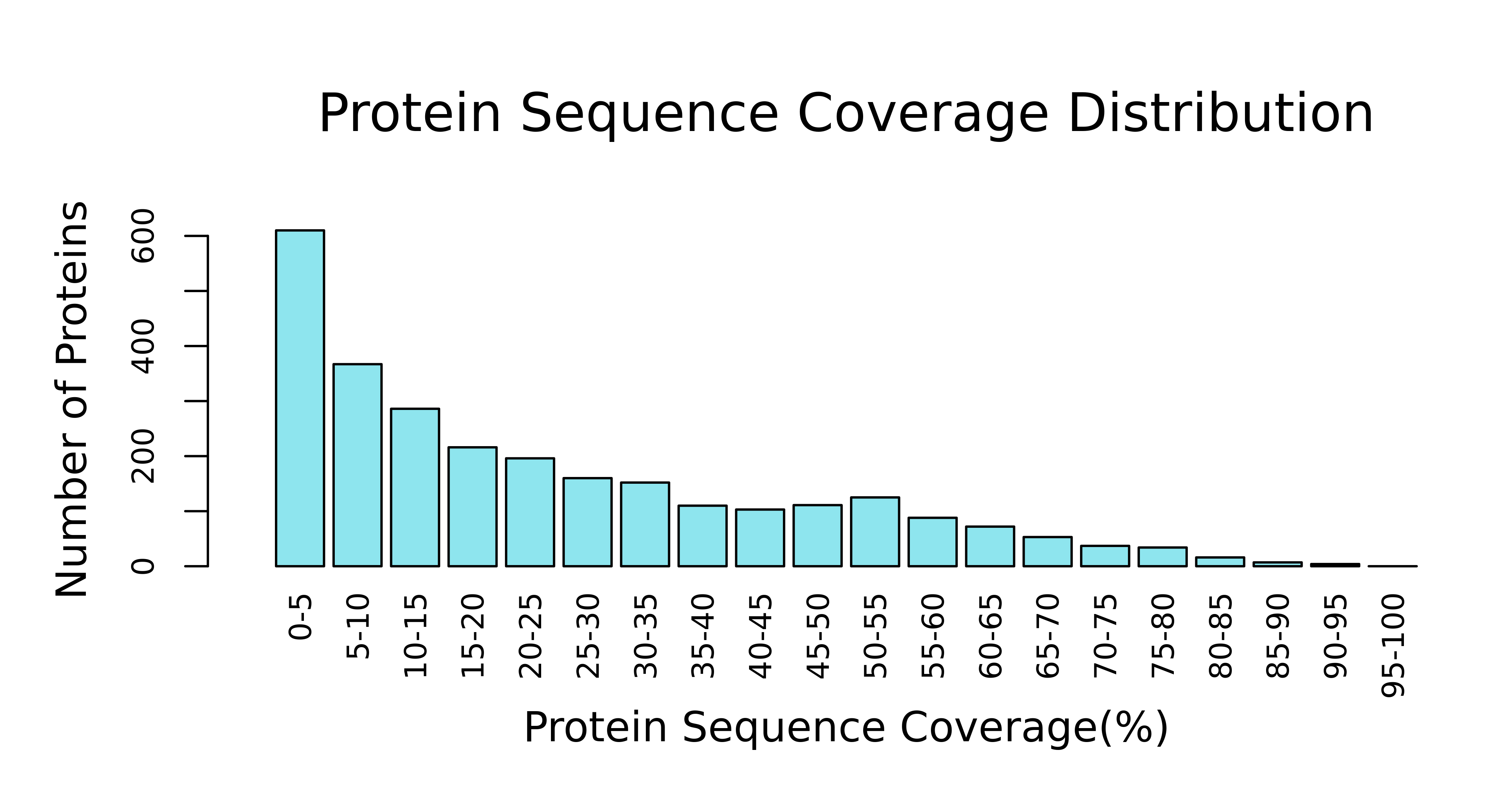


Figure S2.The relationship between molecular weight and coverage of the identified proteins


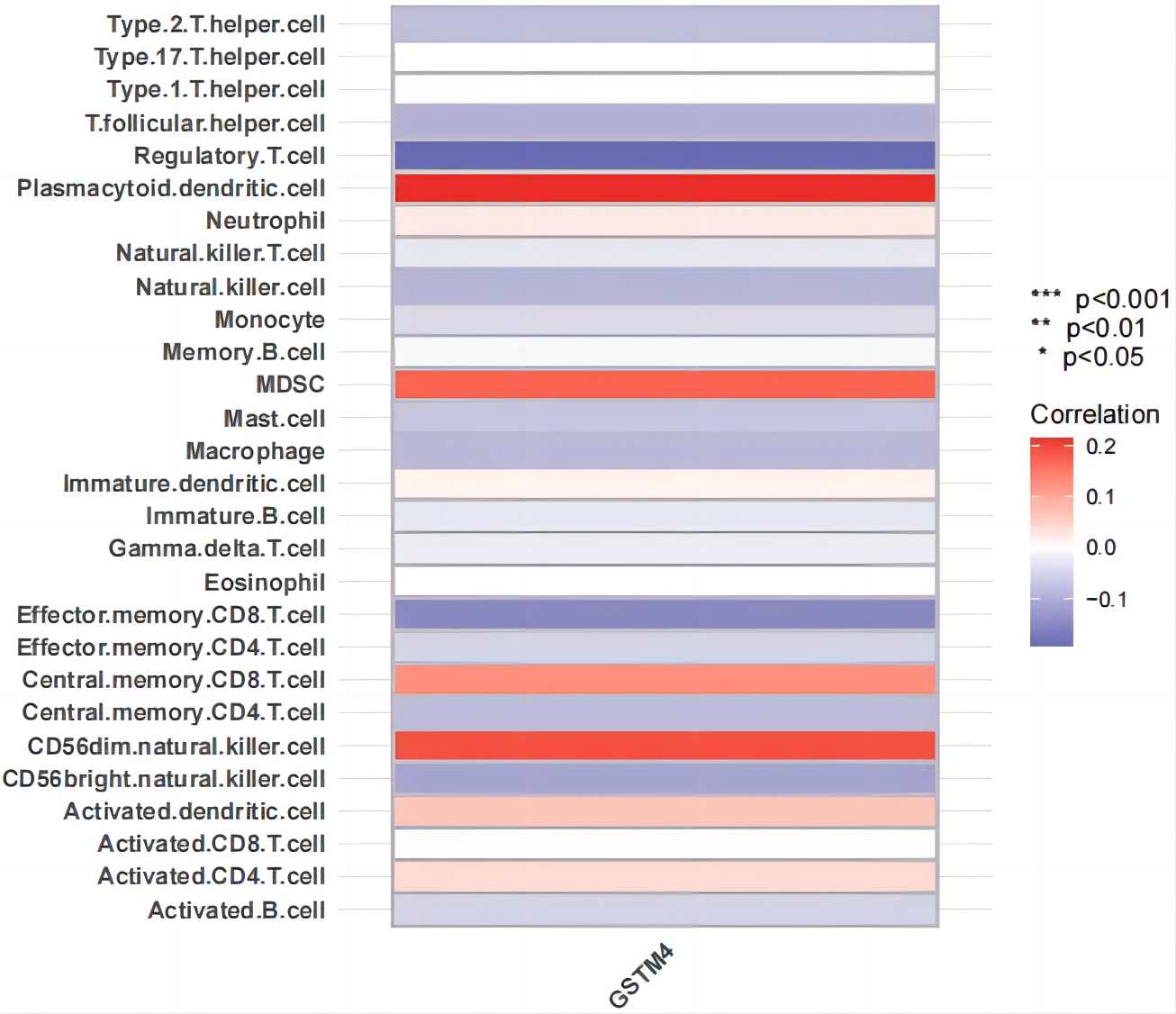


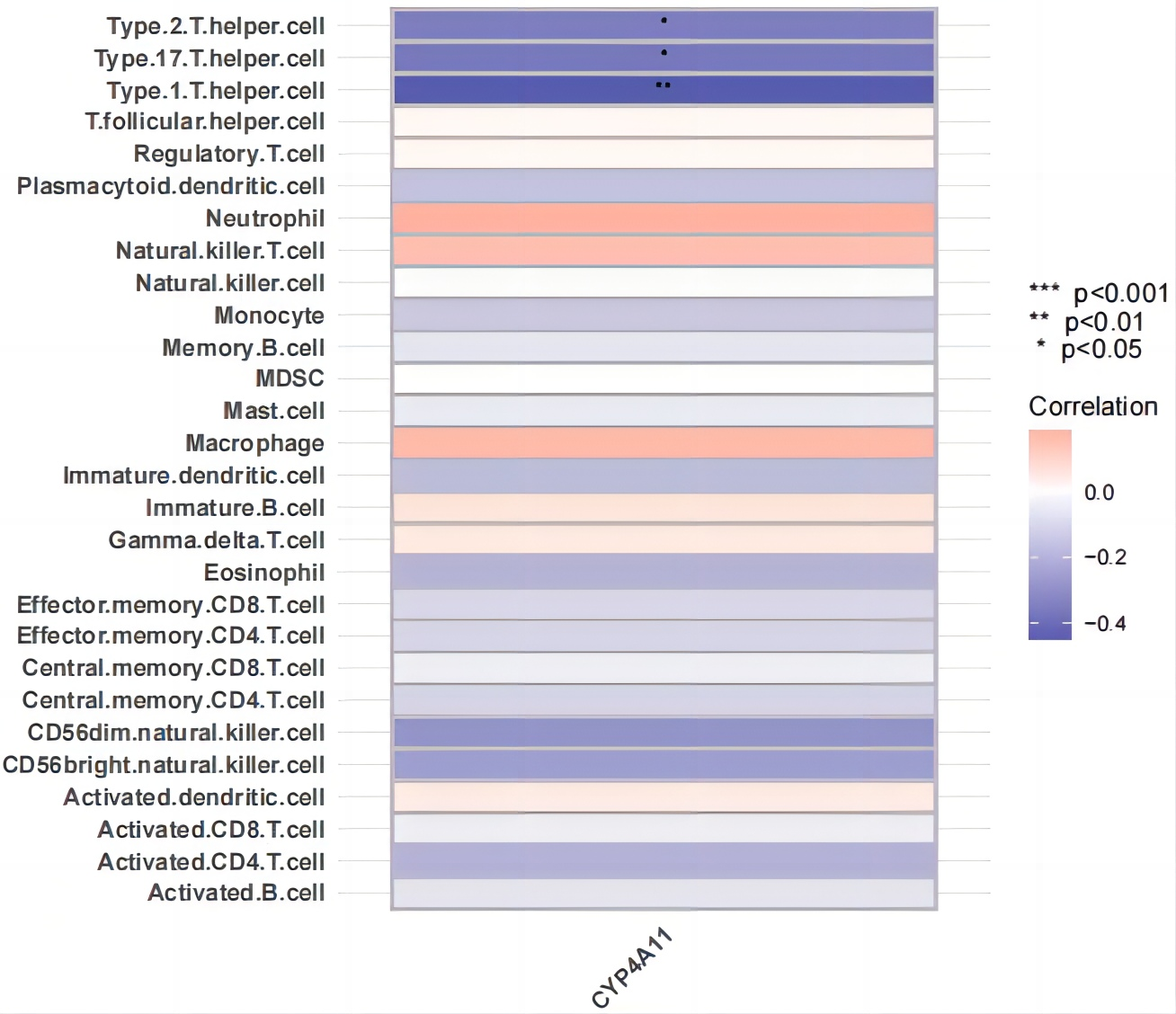


Figure S3.Correlation between CYP4A11 and GSTM4 genes and immune cells.


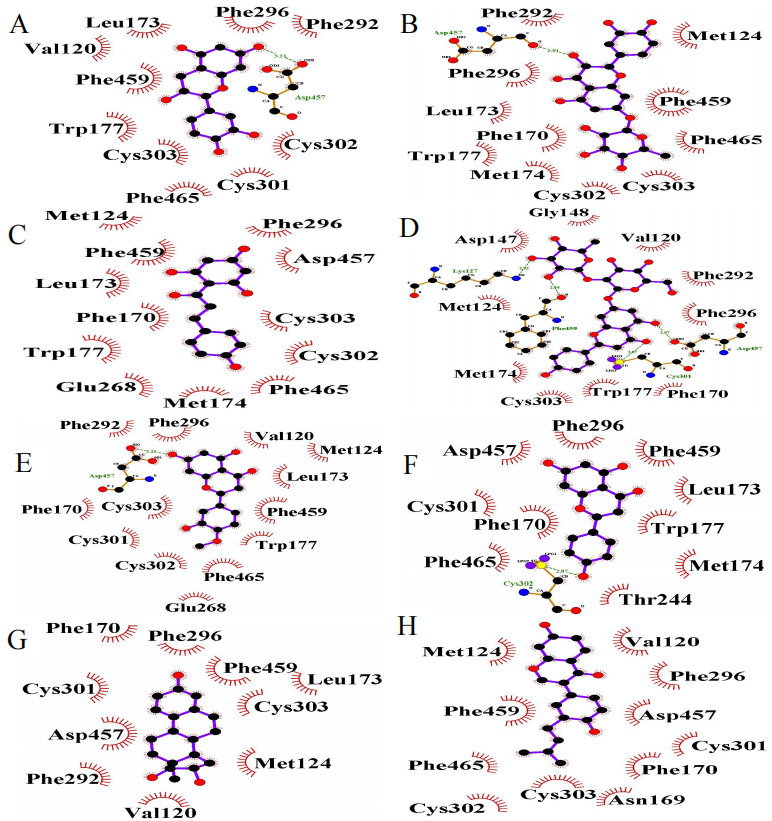


Figure S4.Analysis of hydrogen bond and hydrophobic action. (A)Cianidanol.(B)Taxifolin 7-rhamnoside.(C) Phloretin. (D)Naringin. (E) Hesperetin.(F)Naringenin.(G) Estriol.(H)Neobavaisoflavone.

## Supplementary Tables

Table A1 Database and Software

| **NO.** | **database and software** | **website** |
| --- | --- | --- |
| 1 | GEO (Gene Expression Omnibus) | https://www.ncbi.nlm.nih.gov/geo/ |
| 2 | Funrich3.1.3 | http://www.funrich.org/ |
| 3 | RCSB Protein Data Bank (PDB) | http://www.rcsb.org/pdb |
| 4 | R (4.3.0) | https://cran.r-project.org/bin/windows/base/ |
| 5 | SYBYL 2.1.1 | America Tripos corporation |
| 6 | PubChem | https://pubchem.ncbi.nlm.nih.gov/ |

Table A2 Chromatographic gradient elution program

| Time (min) | Flow rate (μL/min) | A% water | B% acetonitrile |
| --- | --- | --- | --- |
| 0 | 400 | 95 | 5 |
| 3.5 | 400 | 85 | 15 |
| 6.0 | 400 | 70 | 30 |
| 6.5 | 400 | 70 | 30 |
| 12.0 | 400 | 30 | 70 |
| 12.5 | 400 | 30 | 70 |
| 18.0 | 400 | 0 | 100 |
| 22.0 | 400 | 0 | 100 |
| 25.0 | 400 | 0 | 100 |
| 26.0 | 400 | 95 | 5 |
| 30.0 | 400 | 95 | 5 |

Table A3 Changes in blood pressure ( ± *s*, mmHg, 1 mmHg = 0. 133 kPa)

| Group | Systolic blood pressure | | | Diastolic blood pressure | | |
| --- | --- | --- | --- | --- | --- | --- |
|  | 0w | 4w | 8w | 0w | 4w | 8w |
| Control  L-NAME  Captopril  SGR-H  SGR-M  SGR-L | 113.2 ± 4.3  111.3 ± 5.1  118.1 ± 4.6  116.5 ± 6.3  114.7 ± 7.2  115.6 ± 4.9 | 116.5 ± 5.6  151.9 ± 7.1^*^  156.4 ± 5.9^*^  155.3 ± 4.1^*^  154.6 ± 6.9^*^  153.7 ± 7.6^*^ | 115.1 ± 7.1  159.1 ± 6.2*  119.3 ± 5.1^##^  125.6 ± 6.7^##^  132.2 ± 5.6^#^  138.2 ± 6.8^#^ | 93.3 ± 5.1  92.1 ± 4.2  88.9 ± 4.1  89.6 ± 3.6  91.4 ± 4.2  91.6 ± 4.6 | 95.6 ± 4.6  118.1 ± 4.8^*^  117.5 ± 5.8^*^  113.4 ± 3.9^*^  115.7 ± 3.7^*^  112.9 ± 6.3^*^ | 96.3 ± 5.2  117.3 ± 4.5^*^  89.8 ± 4.2^#^  96.8 ± 3.5^#^  100.8 ± 4.8^#^  108.2 ±5.6^#^ |

Note: Compared with the normal control group,* *P* < 0.05, ***P* < 0.01; Compared with the model group (L-NAME), ^#^*P* < 0.05, ^##^*P* < 0.01

Table A4 Detection of serum lipid index（ ± *s*, mmol/L）

| **Group** | **N** | **TC** | **TG** | **LDL-C** | **HDL-C** |
| --- | --- | --- | --- | --- | --- |
| Control  L-NAME  Captopril  SGR-H  SGR-M  SGR-L | 6  6  6  6  6  6 | 2.54±0.56  3.55±0.32^**^  2.98±0.19^#^  2.61±0.42^##^  2.93±0.33^#^  3.50±0.25 | 0.53±0.03  0.83±0.06^**^  0.63±0.03^#^  0.42±0.03^#^  0.58±0.04^#^  0.86±0.02 | 0.52±0.04  0.89±0.05^**^  0.48±0.01^##^  0.62±0.04^#^  0.54±0.02^#^  0.78±0.03 | 1.23±0.04  0.96±0.12^**^  1.67±0.22^##^  1.40±0.24^#^  1.24±0.04^#^  1.17±0.34 |

Note: Compared with the normal control group,* *P* < 0.05, ***P* < 0.05; Compared with the model group (L-NAME), ^#^*P* < 0.05, ^##^*P* < 0.01

Table A5 92 overlapping genes

| **NO.** | **Gene name** | **Gene id** | **NO.** | **Gene name** | **Gene id** |
| --- | --- | --- | --- | --- | --- |
| 1 | COX2 | 4513 | 47 | CYP2C12 | NA |
| 2 | SLC27A2 | 11001 | 48 | ECHDC2 | 55268 |
| 3 | ACOT5 | NA | 49 | IFI47 | NA |
| 4 | AKR1C13 | NA | 50 | GTF2E1 | 2960 |
| 5 | SERPINA3C | NA | 51 | CD68 | 968 |
| 6 | LOC367586 | NA | 52 | CREB3L3 | 84699 |
| 7 | SLC25A20 | 788 | 53 | FAM161B | 145483 |
| 8 | CYP2C13 | NA | 54 | ABAT | 18 |
| 9 | PSMB8 | 5696 | 55 | ADH6 | 130 |
| 10 | LOC100910877 | NA | 56 | ARFGAP3 | 26286 |
| 11 | NQO1 | 1728 | 57 | BYSL | 705 |
| 12 | IGH-6 | NA | 58 | MCPT2 | NA |
| 13 | CES1C | NA | 59 | ARF1 | 375 |
| 14 | CYP4A14 | NA | 60 | GPX7 | 2882 |
| 15 | DPP4 | 1803 | 61 | MSMO1 | 6307 |
| 16 | LYPLAL1 | 127018 | 62 | LAMA2 | 3908 |
| 17 | CROT | 54677 | 63 | CRELD2 | 79174 |
| 18 | CYP3A1 | NA | 64 | CRIP2 | 1397 |
| 19 | MFAP4 | 4239 | 65 | AIF1 | 199 |
| 20 | RT1-E | NA | 66 | IRGM2 | NA |
| 21 | CYP4A2 | NA | 67 | RAB3D | 9545 |
| 22 | PIPOX | 51268 | 68 | RTP4 | 64108 |
| 23 | PDK4 | 5166 | 69 | FGL1 | 2267 |
| 24 | RBKS | 64080 | 70 | ZYX | 7791 |
| 25 | LOC688286 | NA | 71 | FMO5 | 2330 |
| 26 | ADH4 | 127 | 72 | SYAP1 | 94056 |
| 27 | CYP4A12 | NA | 73 | GABARAP | 11337 |
| 28 | TMEM176B | 28959 | 74 | SATB1 | 6304 |
| 29 | AKR1C2 | 1646 | 75 | GSTM4 | 2948 |
| 30 | B3GNT7 | 93010 | 76 | TAP2 | 6891 |
| 31 | PAOX | 196743 | 77 | GNL3L | 54552 |
| 32 | FRMD4B | 23150 | 78 | KRT5 | 3852 |
| 33 | PPIP5K2 | 23262 | 79 | TMEM82 | 388595 |
| 34 | HBB-B1 | NA | 80 | DPYD | 1806 |
| 35 | ACAA1B | NA | 81 | SULT1E1 | 6783 |
| 36 | FBRSL1 | 57666 | 82 | AOX3 | NA |
| 37 | ZFP84 | NA | 83 | KRT73 | 319101 |
| 38 | PAH | 5053 | 84 | DEFA | NA |
| 39 | DHRS7L1 | NA | 85 | CA3 | 761 |
| 40 | CYP8B1 | 1582 | 86 | HP | 3240 |
| 41 | ATP5B | NA | 87 | NUCKS1 | 64710 |
| 42 | REM1 | 28954 | 88 | CES2E | NA |
| 43 | FTCD | 10841 | 89 | ALDH2 | 217 |
| 44 | MAPRE3 | 22924 | 90 | LOC100912026 | NA |
| 45 | GLS2 | 27165 | 91 | RT1-UC | NA |
| 46 | BLVRA | 644 | 92 | MX2 | 4600 |

Table A6 Gene Ontology and KEGG analysis

| **NO.** | **Description** | **Count** | **p-value** | **q-value** |
| --- | --- | --- | --- | --- |
| KEGG | Drug metabolism - cytochrome P450 | 4 | 0.000424499 | 0.027924961 |
|  | beta-Alanine metabolism | 3 | 0.000465416 | 0.027924961 |
|  | Peroxisome | 4 | 0.000728836 | 0.029153445 |
|  | Fatty acid degradation | 3 | 0.001225902 | 0.036777070 |
|  | Pyruvate metabolism | 3 | 0.001588648 | 0.038127550 |
| Cellular Component | peroxisomal matrix | 3 | 0.000597603 | 0.041506746 |
|  | microbody lumen | 3 | 0.000597603 | 0.041506746 |
|  | peroxisome | 4 | 0.001177057 | 0.041506746 |
|  | microbody | 4 | 0.001177057 | 0.041506746 |
| Molecular Function | monooxygenase activity | 5 | 2.52E-05 | 0.003613399 |
|  | oxidoreductase activity, acting on the CH-CH group of donors, NAD or NADP as acceptor | 3 | 0.000112948 | 0.008084682 |
|  | oxidoreductase activity, acting on NAD(P)H | 4 | 0.000212361 | 0.009475677 |
|  | oxidoreductase activity, acting on paired donors, with incorporation or reduction of molecular oxygen | 5 | 0.000325792 | 0.009475677 |
|  | oxidoreductase activity, acting on paired donors, with incorporation or reduction of molecular oxygen, NAD(P)H as one donor, and incorporation of one atom of oxygen | 3 | 0.000330952 | 0.009475677 |
| Biological Process | small molecule catabolic process | 13 | 3.05E-10 | 2.98E-07 |
|  | ethanol metabolic process | 4 | 5.99E-07 | 0.00029293 |
|  | organic acid catabolic process | 8 | 1.51E-06 | 0.000368697 |
|  | carboxylic acid catabolic process | 8 | 1.51E-06 | 0.000368697 |
|  | carboxylic acid biosynthetic process | 8 | 1.12E-05 | 0.001942044 |
